# The toxicological effects of low-density polyethylene microplastic particles (LDPE-MPs) on the growth and metabolic activities of the marine diatom *Chaetoceros muellerii*

**DOI:** 10.1038/s41598-025-27440-9

**Published:** 2025-11-29

**Authors:** Rasha S. Marey, Atef M. Abo-Shady, Hanan M. Khairy, Soha H. Shabaka, Gehan A. Ismail

**Affiliations:** 1 Marine Environment Department, National Institute of Oceanography and Fisheries, NIOF, Cairo, Egypt; 2 Botany Department, Faculty of Science, Tanta University, Tanta, Egypt

**Keywords:** Microplastic pollution, FTIR analysis, SEM images, DSC and combustion techniques, Microplastic adsorption, Hetero-aggregate clusters, Ecology, Ecology, Environmental sciences, Ocean sciences

## Abstract

The aim of the current study is to examine the response of the marine diatom *Chaetoceros muellerii* upon exposure to LDPE-MPs. The toxic effects of LDPE-MP treatment on *C. muellerii* cultures were dependent on its concentration, particle size, and exposure time. The highest percentage of growth inhibition (60.87%) was observed in cultures treated with a dose of 100 mg L⁻^1^ and a particle size of 100 µm of LDPE-MP after 6 days of exposure. A notable reduction was also recorded for the chlorophyll, carotenoids, carbohydrate, and protein contents of the exposed *C. muellerii* cultures compared to the control. In contrast, exposure to LDPE-MPs promoted the lipid content by 47.78 and 51.78% over control at 100 and 250 µm particle sizes, respectively. Also, enhanced the antioxidant activities of CAT (by 41.76 and 33.33%) and SOD (by 57.26 and 44.87%) of *C. muellerii* cultures at both tested particle size, respectively. As a defense mechanism, *C. muellerii* cells secreted exopolysaccharides (EPS) which reached 12.75 and 19.98 folds over control in cultures of 10 mg L⁻^1^ LDPE-MPs at both tested particle size, respectively. The EPS triggered the adsorption of LDPE-MPs on *C. muellerii* surfaces forming hetero-aggregate clusters, obviously shown in the Scanning Electron Microscopy (SEM) images. The Diffraction Scanning Calorimetric (DSC) technique and combustion techniques were applied for quantifying the adsorbed LDPE-MPs on the surfaces of *C. muellerii* cells. The accumulated LDPE-MPs on *C. muellerii* cells at 100 mg L⁻^1^ recorded 0.334 and 0.167 g g^−1^ DW at 100 and 250µm treatment, respectively. To our knowledge, this is the first work to applying both techniques for quantifying MPs accumulated on the microalgal cells, which could be adopted in future studies.

**Supplementary Information:**

The online version contains supplementary material available at 10.1038/s41598-025-27440-9.

## Introduction

Nowadays, plastic plays a crucial role in our daily life by being utilized in household goods, shopping bags, and food packaging^1^. Numerous paths allow large volumes of plastic garbage to reach the environment, where it builds up and poses a major threat to both terrestrial ecosystems and the marine environment^2^. These issues are brought on by the ongoing production of plastics worldwide, a high rate of unintentional discharge, low recycling rates, and insufficient plastic waste management^1^. Plastic wastes enter the environment, and can be transported by wind and water flow and undergo weathering breakdown by physical, chemical, and biological degradation processes^3^. This generates numerous smaller plastic debris, typically less than 1–5 mm in size, which are called microplastic particles (MPs)^4^. MPs have recently been identified as an important emerging worldwide worry that affects aquatic organisms and humans^5^. They showed serious ecological, social, and economic threats, and environmental hazards to ecosystem health^6^. Due to their small size and persistence, MPs can be ingested by marine organisms across various trophic levels. These organisms can either directly uptake free MPs^7^ or indirectly ingest them through the consumption of prey that has been contaminated with MPs^8^. Recent studies confirmed the ability of MPs to pass through biological barriers and bioaccumulate in the organs and tissues, thus induce a wide range of toxic effects on marine organisms of the aquatic food chain^5^^,^^9^.

The low-density polyethylene (LDPE); high-density polyethylene (HDPE), polypropylene (PP), polystyrene (PS), polyethylene terephthalate (PET), polyvinylchloride (PVC), and polyurethane (PU), are the most common and widely produced plastic polymers worldwide^1^^,^^10^. Therefore, they were frequently used as model polymers in the ecotoxicology studies^11^. PE polymer is a simple basic structure consists of long linear hydrocarbon chains of ethylene monomers (C_2_ H_4_)_n_^12^. It has several characteristics that made it attractive for widespread use in industries. PE can be classified into two major groups as LDPE and HDPE based on their density^12^. LDPE is made up of a highly branched hydrocarbon chain, and its low-density characteristic is due to the branching within the polymer backbone. It is resistant to acids, alcohols, bases, and esters but is unstable in the presence of strong oxidizing agents^13^.

Phytoplankton are photosynthetic microorganisms that convert inorganic carbon (CO_2_) into organic carbon source in the form of biomass through photosynthesis^14^. They are important components as primary producers of the trophic chain in the aquatic ecosystems. Therefore, they play a crucial role in maintaining the marine ecosystem balance^15^. Diatoms are considered one of the most diverse and ecologically important phytoplanktonic groups in the marine environment. They may be responsible for about 20% of the overall primary production in the ecosystems and convey a vital role in the biogeochemical cycling of nutrients^16^. Diatoms can attach and colonize to plastics’ surface through electrostatic attraction mechanisms as happen with rocks, sediments, plants, and detritus. The biological adhesion process of diatom to MPs depends on the negatively charged surfaces of the diatom cell^17^. In addition, diatoms can excrete large amounts of transparent exopolymeric substances of highly adhesive properties, which can lead to aggregate formation^18^.

*Chaetoceros* is a common and widely distributed genus of phytoplankton in the marine environments, contributing about 91% of the total phytoplankton cells. Due to its high nutritional values, *Chaetoceros* is used as potential larval feed for shellfish and crustaceans in aquaculture and marine animal hatchery. Colonies of *Chaetoceros* provide an important food source within the water column and are a major carbon contributor to the benthic environment^19^. They have also used as good producers of lipids and other biologically active products, nutraceuticals and sustainable biofuel^20^. *Chaetoceros* species is frequently utilized in photosynthesis experiments and other environmental physiology trials^21^. Additionally, it has been widely used as a typical species for marine ecotoxicological and biotechnological tests due to its small size, high growth rate, and its sensitivity to the environmental stress, including MPs^22^^.^ Therefore, *Chaetoceros* species was selected as a model test-organism for the current study to investigate its response, compatibility, and adaptation to LDPE-MP pollutants.

Eastern Harbor (EH) of Alexandria is a relatively shallow and semi-enclosed bay, covering an area of about 2.6 km^2^. It is situated in the central part of Alexandria coast, Egypt and connected to the Mediterranean open sea through two openings^23^. EH is one of the most important commercial fishing areas on the Mediterranean Coast of Egypt. Currently, EH enclosed extensive discharging of untreated domestic, industrial, and agricultural wastewater and sewage effluents from different sources. Therefore, EH is considered one of the highly polluted and eutrophic area compared to others of the Mediterranean Sea^24^.

Numerous studies have examined the influences and toxicity of MPs on the marine phytoplankton and microalgae species through laboratory experiments^8,12,21^. Still, the MPs’ ecological monitoring could need further investigations, especially in light of the current input of plastics in the environment. Thus, the focus of the present study is to evaluate the potential toxic effects of LDPE-MPs, at different sizes and concentrations, on the growth of the marine diatom *C. muellerii*, based on its actual concentration and properties in the EH area. It is considered the first laboratory study to simulate the natural conditions of MPs (size, composition, and concentration), to which various organisms are exposed in the marine environment. The relationship between particles size, MPs concentration, and toxicity on *C. muellerii* growth and physiological activities, including oxidative stress indices, was evaluated. The study also investigated the possible interaction mechanism between LDPE-MPs and *C. muellerii* cells through visual and instrumental analyses of the DSC- combustion technique and the SEM imaging for detecting the ability of the diatom to accumulate or remove LDPE-MPs from the medium.

## Materials and methods

### *C. muellerii* sampling and culture medium

The marine diatom *C. muellerii* was collected from the surface water of the EH of Alexandria City, Egypt (longitudes 29º53´- 29º54´E and latitudes 31º12´-31º13´N). The samples were collected by using a phytoplankton net of 20 µm pore diameter. In the laboratory, the sampled phytoplankton species were photo-autotrophically grown on the Guillard F/2 enrichment medium^25^. The growth medium was prepared from natural seawater filtered by a sterilized 0.45 µm membrane filter. The solid agar-medium was prepared by adding 1.5% agar to 1 L of the liquid medium, and the pH value of the medium was adjusted at 7 prior to autoclaving.

To obtain a pure culture of *C. muellerii*, the isolation process using Guillard F/2 medium^25^ was carried out by serial agar-plating technique and centrifugation^26^. The collected phytoplankton samples were centrifuged at 4000 rpm for 15 min. The supernatant was decanted, whereas the precipitated algal cells were transferred into a 100 ml glass bottle containing filtered- sterilized seawater enriched with F/2 nutrient medium. The culture bottles were incubated at 25 ± 2 °C under illumination of a 12:12 h light: dark cycle using tubular fluorescent lamps (FL 40 T9D/38) of 3000 lux light intensity. After incubation for 5 days, a sample was transferred to a Sedgewick rafter counting chamber and examined. A single cell of the marine diatom *C. muellerii* was picked up by micropipette under an inverted compound microscope (OPTIKA: XDS-2; Italy). The picked cell was then transferred to a sterile medium on an agar plate that was covered and sealed with parafilm and re-incubated under the previously described conditions. Repeated subculture and microscopic examination of the isolated cells were done on agar medium until obtaining mono (axenic) species of the marine *C. muellerii.* The initial axenic-culture of the diatom was obtained in 250 ml Erlenmeyer flasks, each containing 100 ml of autoclaved F/2 nutrient medium at pH 7^25^. The cultures were incubated at previously described conditions to support algal growth for several days till the exponential growth phase, where they were used as inoculates for the microplastics exposure experiments.

### Identification of the isolated *C. muellerii*

The isolated marine diatom was identified in the laboratory by observing its morphological characteristics under the optical microscope (Model 3B100/ Lamp LED/ DC7.5 W/Germany). Generally, identification of the diatom species was performed according to the following standard keys^27–29^. The diatom isolates were morphologically identified to the species level and subsequently genetically confirmed using 18S rDNA technique^30^.

### Microplastics (MPs) preparation

**Fig. 1:**
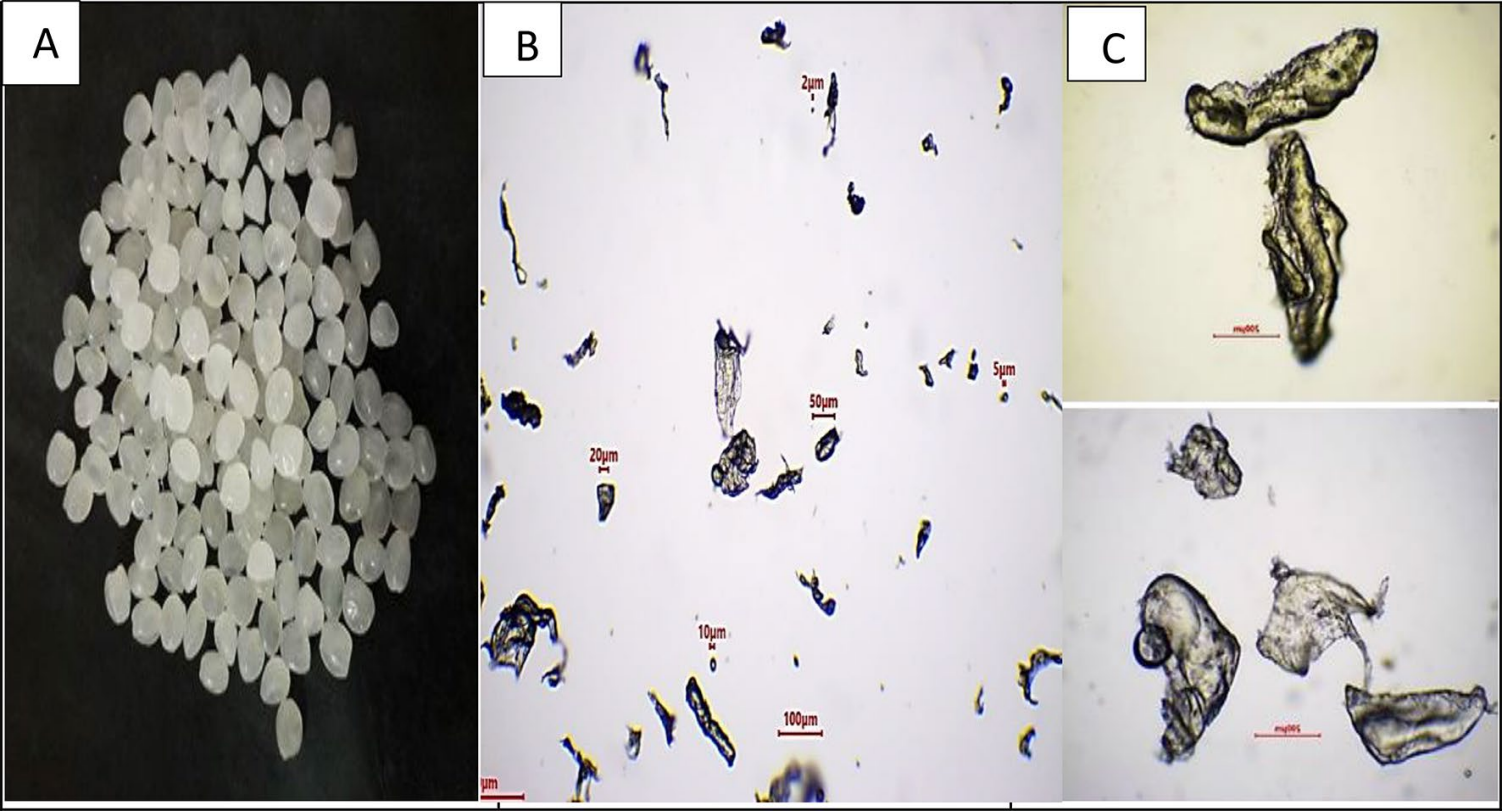
Shape of the LDPE plastic polymer before the fragmentation process (**A**). Shape of the LDPE plastic polymer as appeared under optical microscope after the fragmentation process to form particle sizes of 100 µm at a magnification of 100x** (B**) and 250 µm at a magnification of 200x (**C**)**.**

The commercially manufactured plastic polymer, namely LDPE was used in this study. The polymer was obtained from El SAFWA Company for trading plastic raw materials and high-quality polymers** (**Address: K28-Cairo Alex Desert Road, Abu Rawash Ind. Zone Giza, Egypt). It was described as a pure white spherical pellet with an average diameter of 5 mm (Fig. 1A). The LDPE pellets were further ground by mechanical fragmentation using high-energy milling (2 cycles for 2 min each, at 15 strikes per second) to generate white fine powder of the plastic polymer. The milling process was carried out in a liquid nitrogen atmosphere to decrease polymer elasticity. Generated fragments were separated into two size ranges using two stainless steel sieves of 1–100 and 100-250 µm mesh sizes. These two sizes were the most widely sampled from the typical environment of the EH Bay in a previous study^31^. After the fragmentation process, the LDPE-MPs were washed with 70% ethanol to clean any contaminants on the plastic and dried at room temperature for 24 h.

### Microplastics characterization

LDPE-MPs were observed by the optical microscopy at 40 × magnification to describe their morphological characteristics, including shape, size, and color. White, irregular plastic particles with sizes ranging from 1 to 250 µm in diameter were observed (Fig. 1 B and C). The chemical nature of the LDPE-MPs was confirmed by the DSC technique^31^ and had a specific density of 0.923 g cm^−3^. The integrated selection of the polymer was based on the characterization and quantification results of MPs assessed previously by Shabaka et al.^31^ in the area of EH Bay.

### Toxicological experiment setup

#### Exposure of *C. muellerii* to LDPE-MPs

The size and concentration effects of LDPE-MPs were studied in relation to the growth, photosynthesis, proximate contents, and oxidative stress parameters of *C. muellerii *cultures. Two different sizes of 1–100 and 100–250 µm were used for the selected LDPE-MPs polymer. For each tested size, four concentrations of 10, 25, 50, and 100 mg L^−1^ of diatom culture were applied. It should be noted that this set of LDPE-MP concentrations was higher than that in situ reported in the marine waters of the EH area to test the maximum microplastic concentration that can be afforded by *C. muellerii.*

#### Culture conditions and diatom growth

**Fig. 2:**
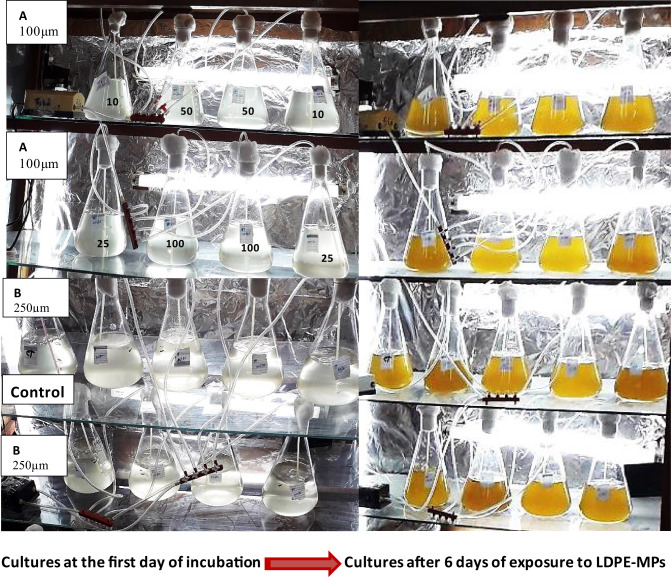
Cultures of the marine diatom *C. muellerii* before and after exposed to LDPE-MPs for 6 days at different particle size and concentrations; Control culture is without LDPE-MP addition.

*C. muellerii *was cultured photo-autotrophically on F/2 enrichment medium^25^ under axenic conditions (Fig. 2). All cultures were grown in 1000 ml Erlenmeyer flasks containing 800 ml of the autoclaved nutrient medium. The exponentially grown algal cultures (after 6 days) were used as an inoculum for all experiments. The initial optical density (OD_680_) was 0.02 at 680 nm, and the initial cell density was 4.8 × 10^7^cells ml^−1^ at the start of the experiment. LDPE-MP was directly added to the culture medium at concentrations of 10, 25, 50, and 100 mg L^−1^. A culture growth medium of *C. muellerii *without added LDPE-MPs was used as a control, and each experimental treatment was performed in triplicate. After the MPs addition, the cultures were gently mixed on a shaker, achieving a homogenous distribution for LDPE-MPs into the diatom growth culture. This method of dispersion was a more realistic representation of natural conditions for MPs in the marine environment^32^. All cultures were then incubated for 6 days under the same previously mentioned conditions. To avoid settling, accelerating the growth process, and to provide necessary CO_2,_ continuous aeration was supplied to the culture by bubbling filter-sterilized air. Besides, the cultures were well shaken manually three times a day. During the experimental period, the changes in OD_680_ and cell counting of *C. muellerii, *in addition to the quantity of the photosynthetic pigments, were recorded every 24 h as growth indices.

### Assessment of *C. muellerii* growth parameters under LDPE-MPs exposure

#### Optical density

The OD_680_ of *C. muellerii *was determined daily by absorbance measurement of the cultures at 680 nm wavelength^33^ using a UNICO UV/Visible spectrophotometer (model 2000 UV, power source, AC220V/50HZ). The corresponding growth curve of the treated and control cultures of *C. muellerii *were developed by plotting the OD_680_ value against the incubation time of the culture.

#### Cell counting and growth rate

The cellular densities of the control and treated cultures were measured by counting the *C. muellerii *cells under the optical microscope with 40 × magnification using a hemocytometer cell counting chamber (Improved Neubauer, CHINA). The cell density was estimated every 24 h throughout the period of the experiment (6 days), and the growth curve was recorded. The number of cells per ml was evaluated, and the specific growth rate (μ) was calculated according to following Eq. ^34^

$$\mu \left( {{\text{day}}^{{ - {1}}} } \right) = {\text{ln }}\left( {{\text{N}}_{{2}} - {\text{N}}_{{1}} } \right)/({\text{T}}_{{2}} - {\text{T}}_{{1}} )$$

Where μ is the specific growth rate, N_2_ is the total cell count (mL^−1^) at the final day (i.e., the end of the exponential growth phase, T_2_), and N_1_ was the total cell count (mL^−1^) at the initial day (the beginning of the exponential growth phase, T_1_).

In addition, the growth inhibition ratio (IR) of the *C. muellerii *cultures was calculated according to following Eq. ^34^

$${\text{IR}}\left( \% \right) = {\text{Cci}}{-}{\text{Cei}}/{\text{Cci}} \times {1}00$$

Where IR is the growth inhibition rate at time i; Cci is the cell number of the control algal culture at time i; and Cei is the cell number of the treated culture at time i.

#### Dry weight (DW)

The DW of *C. muellerii *cultures at different MP concentrations was estimated by centrifugation (Fisher Centerific ^Tm^ Centrifuge) of a known culture volume (50 ml) at 3000 rpm for 10 min. The cell pellets were washed twice using distilled water and then oven- dried at 80 °C in pre-weighed tubes using an analytical balance (± 0.0001 g) until constant weight. The recovered biomass was expressed as milligrams per liter (mg L^−1^) of the culture.

#### Pigments involved in photosynthetic processes (carotenoids, chlorophyll-a, and c)

Every day during the MPs exposure period of *C. muellerii*, the photosynthetic pigments were measured using a spectrophotometric technique. To get biomass, a known volume of the culture was pipetted out and centrifuged for 10 min at 3000 rpm. The 100% methanol approach was used to extract the pigments from the biomass^35^. A Vis-spectrophotometer was used to measure the absorbance values for carotenoids at 450 nm and for chlorophyll a and chlorophyll c pigments at 664, 647, and 630 nm. Chlorophyll a and c concentrations were calculated as µg/mL of algal suspension^36^

$${\text{Chlorophyll}}a\, = \,{11}.{85 }\left( {{\text{Abs}}_{{{664}}} } \right){-}{1}.{54 }\left( {{\text{Abs}}_{{{647}}} } \right){-}0.0{8 }({\text{Abs}}_{{{63}0}} )$$

$${\text{Chlorophyll}}c\, = \,{24}.{52 }\left( {{\text{Abs}}_{{{63}0}} } \right) \, {-}{7}.{6}0 \, \left( {{\text{Abs}}_{{{647}}} } \right){-}{1}.{67}({\text{Abs}}_{{{664}}} )$$

The carotenoids content (µg/mL) was calculated using the equation of Prieto et al.^37^ as: Carotenoids = 25.2 (Abs _450_).

### Estimation of the biochemical compositions of *C. muellerii* cultures under LDPE-MPs exposure

#### Total soluble protein content

A known volume (5 ml) of *C. muellerii *culture was centrifuged at 3000 rpm for 10 min. The supernatant was decanted, and 5 ml of sodium hydroxide (NaOH, 1 N) was added to the pellet in a boiling water bath for 2 h for protein extraction^38^. The protein content was determined according to the Bradford method^39^ at 595 nm. A standard curve of bovine serum albumin was used as a reference and the protein content was expressed as mg/g DW of the pellet.

#### Total soluble carbohydrate and exopolysaccharides (EPS) contents

The total carbohydrate content was quantitatively determined for *C. muellerii* biomass in the same previous NaOH extract using the phenol- sulfuric acid (H_2_SO_4,_ 95.5%) method^40^. Main while, the cell-free supernatant of the culture was used to estimate the dissolved exopolysaccharides (EPS). To precipitate the EPS from the supernatant, 95% cold ethanol was added to the supernatant, typically a 2- to 3- fold of the supernatant volume, and incubated at 4 ℃ overnight for precipitation. Then, the precipitated EPS was recovered by centrifugation, washed and resuspended in a propriate volume of deionized water. Finally, the content of the extracted EPS was quantified using the same method. The absorbance of all samples was measured at 490 nm against blank. The carbohydrates and EPS concentration of the samples were estimated from a calibration standard curve of glucose.

#### Total lipids content

The extraction of lipids was done by the modified Folch method^41^. Briefly, 20 ml of *C. muellerii *culture were collected and centrifuged at 3000 rpm for 10 min. the supernatant was discarded, and 10 ml of chloroform/methanol (2/1,* v/v*) were added to the pellet cells and extracted for 2 h in a shaker. The glass vials containing the lipid extracts were dried at 80 °C for 30 min, cooled in desiccators, and weighted. The lipid content was calculated as mg g^−1^ DW of the algal cells (pellet).

### Estimation of the antioxidant enzymes (Oxidative stress) of *C. muellerii* cultures under LDPE-MPs exposure

To evaluate the toxic effects (oxidative stress) of LDPE-MP on *C. muellerii *cells, the activities of the antioxidant enzymes involved in scavenging reactive oxygen species, viz., superoxide dismutase (SOD) and catalase (CAT), were assayed after 6 days of MP exposure as follow:

#### Extraction of the enzymes

To extract the antioxidant enzymes, a known volume of each culture was centrifuged at 4000 rpm for 10 min, and the supernatant was removed. A known fresh weight (0.5 g) of the diatom cell pellets was frozen, then homogenized in 10 ml of 100 mM phosphate buffer (KH_2_PO_4_/ K_2_HPO_4_) with a concentration of 19.5 ml/30.5 ml, pH 7.0, containing 0.1 mM of Na_2_ EDTA and 0.1 g of polyvinyl pyrrolidone^42^. The homogenate was centrifuged at 4000 rpm at 4 °C for 10 min. The supernatant was collected and stored at 4 °C in the dark for the enzymatic assay.

#### Assay of catalase

CAT (EC1.11.1.6) activity was assayed by measuring the initial rate of hydrogen peroxide (H_2_O_2_) disappearance or consumption^43^. The assay medium contained 1.5 ml of 0.1 M sodium phosphate buffer (pH 7), 0.3 ml of 2 mM H_2_O_2_ and 0.1 ml of the enzyme extract. Then 1.1 ml of distilled water was added up to 3 ml final assay volume. The decrease in H_2_O_2_ was monitored at 240 nm using a Vis-spectrophotometer. CAT activity was calculated using an extinction coefficient of 40 Mm^−1^ cm^− 1^ at 240 nm and expressed in units of μM H_2_O_2_ converted per minute per gram fresh weight of the algal pellet (min^.−1^ g^−1^ FW).

#### Assay of superoxide dismutase

The principle of SOD (EC 1.151.1) activity is based on the reduction inhibition of nitro blue tetrazolium (NBT) reagent, as indicated by the method of Beyer and Fridovich^44^. The reduction of NBT by superoxide radicals to blue color formazan was followed at 560 nm. The reaction mixture was prepared by mixing 27 ml of 50 mmol/l potassium phosphate buffer (pH 7.8), 1.5 ml of L-methionine (300 mg/10 ml), 1 ml of NBT salt (14.4 mg/10 ml), and 0.75 ml of Triton X-100. Aliquots (1 ml) of the mixture was delivered into a small glass tube, followed by 20 μl of the enzyme extract and 10 μl of riboflavin (4.4 mg/ 100 ml). In the control tube, the sample was substituted by 20 μl of the buffer. The cocktail was mixed in glass tubes, immersed in a cylindrical glass container filled with three-fourth clean water, then illuminated by two 20W fluorescent lambs at 25 °C for 7 min. The increase in absorbance due to formazan formation was read at 560 nm. Under the described conditions, the increase in absorbance in the absence of the enzyme extract was taken as 100%. The enzyme activity was calculated by determining the percent inhibition per min. Fifty percent inhibition was taken as equivalent to 1 unit of SOD activity.

### Microscopic examination of *C. muellerii* cultures under LDPE-MPs exposure

#### Optical microscopy

The microscopic analyses and photo imaging of *C. muellerii *cells exposed to LDPE-MP were carried out using the optical microscope (Model 3B100, LED Lamp DC7.5 W, Germany). The images were taken with a digital camera (5.1MP C- Mount Camera, GZM-TR-745) at 40 × magnification using Pico Stage (× 86) Software. Samples were left to settle for about 20 min before the microscopic observations. Then, a glass Pasteur pipette was used to draw small amount of the sample from the bottom of the vial. The drawn sample was put on a clean microscopic slide, covered by glass cover, and then examined. 

#### SEM image

The interaction between the LDPE-MP and *C. muellerii* cells was observed after 6 days of exposure using SEM technique. To prepare samples, the cells were centrifuged, and the pellet cells were fixed by 2.5% glutaraldehyde at 4 °C overnight. The fixed cells were then washed three times with 0.1 M phosphate buffer solution (pH 7.4). Then, the cells were fixed by 1% osmium tetroxide at 4 °C for 1 h and washed three times with the buffer followed by centrifugation. The fixed cells were then dehydrated successively through a series of alcohol solutions for 20 min. After that, samples were fixed with tert- butyl alcohol and later subjected to freeze- drying. At last, samples were metallized and observed using SEM instrument (JSM-IT200 Series in Touch Scope™ Scanning Electron Microscope JEOL).

### Qualitative and quantitative estimation of adsorbed LDPE-MP on *C. muellerii* cells

#### Harvesting of the algal biomass

After 6 days exposure to the LDPE-MPs, the biomass of *C. muellerii *was harvested from the cultures using the sedimentation methods. Cultures were transferred to a refrigerator and allowed to settle at the bottom of the flask for 48 h. The floated portion of the culture medium containing the residual LDPE-MPs was withdrawn using a siphoning technique. The settled biomass was centrifuged at 3000 rpm for 10 min. Finally, the wet *C. muellerii *biomass was dried in an oven at 40 °C for 24 h and kept in clean vials for further analysis.

#### Fourier transform infrared spectroscopy (FTIR) analysis

FTIR analysis was conducted using the dried biomass of *C. muellerii. *The purpose of the FTIR test was to study the chemical bonding of the diatom biomass and to determine the effect of LDPE-MPs on the variations of the functional organic groups in *C. muellerii* cells. The method practically involves making small salt discs composed of 1 mg of the tested material (dried algal biomass) and 300 mg of pure dry KBr, which were pressed mechanically into a disc. The disc was then measured using Perkin Elmer 1430 spectrophotometer at infra-red spectra range from 500 to 4000 cm^−1^ as described by Robyte and White^45^.

#### Thermal analysis

Thermo-analytical techniques include different systems such as DSC, thermogravimetry (TGA), and pyrolysis–gas chromatography-mass spectrometry (py-GC–MS). In this methodological approach, two thermal analyses were integrated for the first time. The DSC (model Q2000 V24.11, build 124) was employed to qualitatively examine the LDPE-MP polymer type and its aggregated quantity on the surface of *C. muellerii* cells^31,46^. The combustion technique was used for estimating the quantity of LDPE-MPs aggregated on the surface of *C. muellerii* cells. The quantification was conducted by recording the percentage of weight loss according to thermal decomposition temperatures of the sample components using a laboratory muffle furnace (Nabertherm, Germany)^46^. According to this technique, three main thermal decomposition stages were involved in the combustion of a specific organic sample. The first stage was at 150 °C, mainly caused by volatilization of the water content. The second stage was at 300 °C due to the decomposition of organic matter or natural polymers such as chitin, chitosan, and cellulose. The third decomposition temperature was at 450 °C due to combustible material such as plastics polymers. Basically, the largest mass changes at 330 °C and 460 °C were due to MPs content^47^. All the experimental procedures for LDPE-MPs and *C. muellerii* cultures were consistent with the quality control assessment protocols^48^ to prevent contamination during laboratory practices and validate the accuracy of the results.

#### Statistical analysis

The results were expressed as the mean of three experimental replicates ± standard deviation (SD). The statistical analysis was carried out using SPSS statistical package (IBM, Version 22.0, 2013). The obtained data was statistically analyzed to evaluate the degree of significant differences between the control (untreated) algal cultures and the LDPE-MP treated cultures at different sizes and concentrations of the polymers using one-way analysis of variance (ANOVA) at *p* ≤ 0.05.

## Results

### Identification of the isolated diatom species *C. muellerii*

#### Morphological identification

**Fig. 3:**
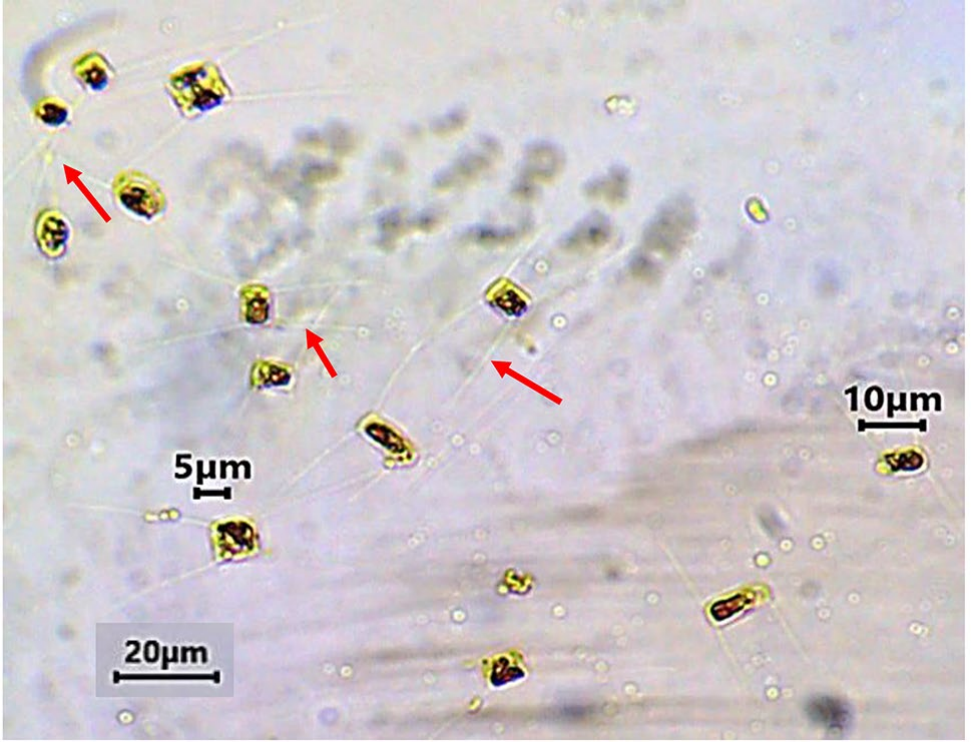
Cell structure of the marine planktonic diatom *C. muellerii* isolated from seawater of Eastern Harbor Bay, Alexandria, and grown in F2/enrichment culture medium at a magnification of 200x. *Note* that individual cell walls are composed of silica that contain long and thin spines (setae) marked with red arrows. The spines may connect the frustules together forming a colony of multiple cells.

The morphological identification of the isolated marine diatom by light microscope revealed that the strain had consistent morphology to *C. muellerii,* which is a unicellular centric marine diatom belongs to Bacillariophyceae taxonomic group. As a diatom, it represents the first trophic level of the marine aquatic food chain. It is a non-motile cell with a 4 µm width and 7 µm length enclosed in siliceous valves known as frustules or cell walls that are composed of silica covered with an organic coating (Fig. 3). Besides, *C. muellerii* has four long, thin spines (setae) that extend from corners of their frustules. The spines connect the frustules or multiple cells together, creating a colony of cells. Colonies, in turn, can form chains that are coiled, straight, or curved. This identification was subsequently confirmed by 18S rDNA molecular identification.

#### Molecular identification

The 18S rRNA gene sequences of the isolated marine diatom showed that the base size of *C. muellerii* was 858 bp. The sequences results for this isolate indicated a 100% similarity to the 18S ribosomal RNA sequence of *C. muellerii* strain Xiamen Harbor, which includes the partial sequence of the 18S ribosomal RNA gene, internal transcribed spacer 1, the complete sequence of the 5.8S ribosomal RNA gene, internal transcribed spacer 2, and the partial sequence of the 28S ribosomal RNA gene. After sequencing, these rRNA gene sequences were registered in a DNA database with accession number KF998567.1 (Table 1 and Fig. 1S).

**Table 1:** Accession number of *C. muellerii* strain at the gene bank by using 18S rRNA gene sequence.

GenBank	Highest homology identification	Identification
KF998567.1	*Chaetoceros muellerii* strain Xiamen Harbor 18S ribosomal RNA gene, partial sequence; internal transcribed spacer 1, 5.8S ribosomal RNA gene, and internal transcribed spacer 2, complete sequence; and 28S ribosomal RNA gene, partial sequence	100%

### Growth curve and cell density of *C. muellerii* in the control cultures

Three phases of *C. muellerii* growth curve were observed, the exponential phase (log phase), the stationary phase, and the death phase, while the lag phase was not detected (Fig. 2SA). The results showed that the highest growth of *C. muellerii* (OD_680_ value of 0.633) was obtained at the 6^th^ day of incubation. A rapid increase in the cell division of *C. muellerii* was noticed during six days of the exponential growth phase, followed by constant increase in the stationary phase, then gradual decrease for ten days. The same growth pattern was observed with the cell density expressed as cell count (Fig. 2SB). The highest cell density of* C. muellerii* was 1240 × 10^6^ cells mL^−1^, recorded at the late exponential growth phase, and the lowest cell count was 196 × 10^6^ cells mL^−1^, recorded at the end of the growth curve.

### Indices of *C. muellerii* growth under LDPE-MPs treatment

#### Optical density

The OD_680_ curves showed that LDPE-MPs had a slight inhibitory effect on the growth of *C. muellerii* at all-tested concentrations, in comparison with the control group. The growth was obviously decreased with the increase of LDPE-MPs concentration in the culture medium (Fig. 3S). The cultures treated with LDPE-MPs, of 100 µm particle size, showed a decrease in the OD_680_ value by 7.02, 11.65, 17.01, and 25.32%, at different concentrations of 10, 25, 50, and 100 mg L^−1^, respectively, during 6 days of MPs exposure (Fig. 3SA). The same pattern of growth was observed with *C. muellerii* cells exposed to 250µm particle size of LDPE-MPs, which resulted in the reduction of OD_680_ value by 4.25, 7.21, 14.79, and 21.99% at the same examined concentrations, respectively (Fig. 3SB).

#### Cell counting

**Fig. 4:**
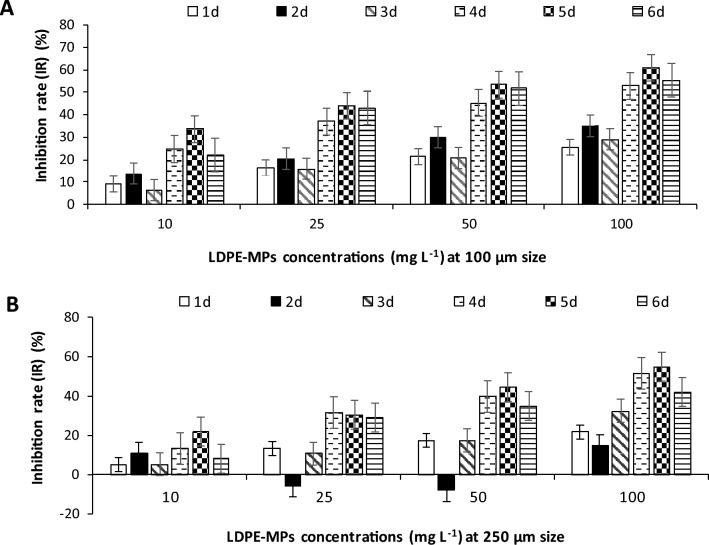
Growth inhibition rate of *C. muellerii* cultures exposed to LDPE-MPs (mg L^−1^) with particle size of 100 µm** (**A) and 250 µm (B) during 6 days of incubation.

The effect of LDPE-MPs treatment on *C. muellerii* cell number as indicative of growth was dependent on the particle size, particle concentration, and treatment (exposure) time. The cell number of *C. muellerii* decreased with the increase in LDPE-MPs concentration in the culture medium. Using LDPE-MP of 100µm particle size gradually reduced the cell number by 22.23, 43.17, 51.75, and 55.31% at the doses of 10, 25, 50, and 100 mg L^−1^, respectively compared to the control culture (Fig. 4SA). Similarly, the cultures exposed to 250µm LDPE-MP resulted in decreased cell number by 7.97, 28.91, 34.75, and 41.88% at the same tested concentrations, respectively (Fig. 4SB). The minimum cell number of 589 × 10^6^ cells ml^−1^ was recorded for LDPE-MPs of 100µm particle size and 100 mg L^−1^ dose after 6 days of exposure.

#### Growth rate

The inhibition effect of LDPE-MPs on the growth of *C. muellerii* cells was dependent on the concentration and exposure time. The maximum growth inhibition rates of 60.87% and 54.65% were recorded in *C. muellerii* cultures exposed to 100 mg L^−1^ dose of LDPE-MPs at 100 and 250µm particle size, respectively, on the 5^th^ day of exposure (Fig. 4). However, the inhibition of growth rate was notably decreased at the 6^th^ day for all tested concentrations using both particle sizes. It was noticed that an enhancement in the growth rate was recorded on the 2^nd^ day of exposure at 25 and 50 mg L^−1^ for LDPE-MP of 250µm particle size (Fig. 4).

**Fig. 5:**
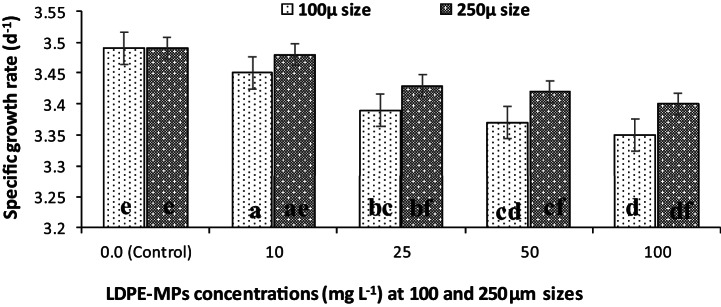
Specific growth rate (d^−1^) of *C. muellerii* cultures exposed to LDPE-MPs (mg L^−1^) with particle size of 100 and 250 µm during 6 days of incubation. Columns with different letters indicated significant difference between the polymer concentrations at *P* ≤ 0.05.

In addition, negative effects of LDPE-MPs on the specific growth rate (µ) of *C. muellerii* cultures per day were estimated at all tested concentrations (Fig. 5). The µ values were gradually decreased with increasing LDPE-MP concentrations for 100 and 250µm particle size, compared to the µ values of the control cultures (0 mg L^−1^ LDPE-MPs). Therefore, the highest value of µ (3.49 d^−1^) was recorded for the control cultures.

### Effect of LDPE-MPs treatment on the photosynthetic pigments of* C. muellerii* cultures

#### Chlorophyll *a *(Chl *a*) content

The Chl *a* content was extremely reduced by increasing LDPE concentration in the culture medium of *C. muellerii*. By using 100µm LDPE-MPs treatment, a significant (*p* ≤ 0.05) gradual decrease by 5.44, 13.07, 19.31, and 22.96%, was recorded, compared to the control cultures at 10, 25, 50 and 100 mg L^−1^ of LDPE-MPs, respectively (Fig. 5SA). A similar significant inhibition in Chl *a* content was recorded to be 1.63, 7.66, 11.73, and 17.36%, for cultures treated with 250µm LDPE-MPs at the same previous concentrations, respectively (Fig. 5SB). The lowest content of Chl. *a* (2.876 µg ml^−1^) was obtained at LDPE-MPs of 100 mg L^−1^ dose and a 100µm particle size after 6 days of exposure.

#### Chlorophyll *c *(Chl *c*) content

**Fig. 6:**
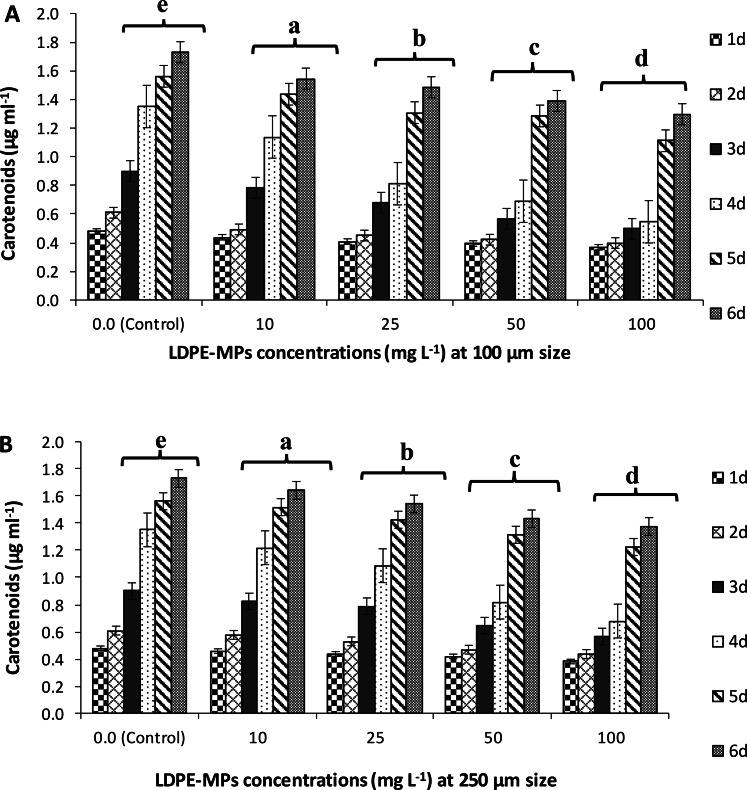
Carotenoid content (µg ml^−1^) of *C. muellerii* cultures exposed to LDPE-MPs (mg L^−1^) with particle size of 100 µm** (A**) and 250 µm (**B**) during 6 days of incubation. Error bars showed the SD for three replicates. Columns with different letters showed significant differences between polymer’ concentrations compared to the control (cultures without LDPE-MPs treatment) at *P* ≤ 0.05.

The results showed that higher LDPE-MP concentrations in the culture medium caused a higher reduction percentage of Chl *c* content compared to the control cultures. *C. muellerii* cultures treated with 100µm particle size resulted in a significant (*p* ≤ 0.05) inhibition of Chl *c* content by 16.04, 21.93, 27.60, and 30.59% at LDPE-MP concentrations of 10, 25, 50 and 100 mg L^−1^, respectively (Fig. 6SA). Similarly, Chl *c* content was significantly mitigated by 7.65, 11.96, 16.31, and 23.12% when the cultures were provided with 250µm particle size at the same previous concentrations of LDPE-MPs, respectively (Fig. 6SB). The minimum content of Chl *c* (1.579 µg ml^−1^) was estimated for* C. muellerii* cultures treated with 100 mg L^−1^ dose and a 100µm particle size of LDPE-MPs after 6 days of exposure.

#### Carotenoid content

By using LDPE-MPs of 100µm, a significant (*p* ≤ 0.05) reduction percentage of 10.64, 14.11, 19.66, and 24.99% was recorded for the carotenoid content at 10, 25, 50, and 100 mg L^−1^, respectively (Fig. 6A). A similar pattern was found when *C. muellerii* cultures were exposed to 250µm LDPE-MPs, which resulted in 5.03, 10.82, 17.18, and 20.42% decrement in the carotenoid content of *C. muellerii* at the same concentrations, respectively (Fig. 6B). The lowest content of carotenoid (1.297 µg ml^−1^) was estimated at LDPE-MPs treatment dose of 100 mg L^−1^ and a 100µm size after 6 days of exposure.

### Effect of LDPE-MPs treatment on the biochemical compositions of *C. muellerii* cultures

#### Total protein content

The effect of LDPE-MP concentrations (at 100 and 250µm particle sizes) on the total protein content of *C. muellerii* cultures was shown in Table 2. A significant (*p* ≤ 0.05) decrease in the protein content by 9.76, 15.07, 22.90, and 27.26% compared to the control was estimated for the cultures treated with 100µm of LDPE-MPs at 10, 25, 50 and 100 mg L^−1^, respectively, after 6 days of exposure. In the same way, significant reduction percentages in the protein content by 4.52, 12.63, 17.78 and 23.96% were evaluated for the cultures exposed to 250 µm particle size at the previous LDPE-MP concentrations, respectively (Table 2). The minimum protein content of 119.60 mg g^−1^ DW was recorded at LDPE-MPs of 100 mg L^−1^ dose and 100µm particle size.

**Table 2:** Proximate and EPS contents of *C. muellerii* cultures treated with 100 and 250µm particle sizes of LDPE-MPs at different concentrations (mg L^−1^) after 6 days of culture exposure*

LDPE-MPs (mg L^−1^) at 100 µm size	Proximate compounds (mg g^−1^ DW)			EPS content (mg L^−1^)
	Carbohydrates	Proteins	Lipid content	
**0 (Control)**	76.51 ± 0.61^e^	164.43 ± 2.25^e^	364.4 ± 4.23^e^	1.67 ± 0.22^e^
**10**	67.33 ± 0.70^d^	148.38 ± 5.49^d^	385.4 ± 4.89^d^	21.29 ± 0.25^a^
**25**	50.55 ± 1.27^c^	139.65 ± 5.46^c^	428.6 ± 4.64^c^	14.76 ± 0.22^b^
**50**	42.25 ± 1.09^b^	126.77 ± 1.54^b^	484.3 ± 5.10^b^	9.62 ± 0.36^c^
**100**	34.78 ± 0.94^a^	119.60 ± 4.92^ab^	538.5 ± 6.90^a^	3.33 ± 0.22^d^

LDPE-MPs (mg L^−1^) at 250 µm size	Proximate compounds (mg g^−1^ DW)			EPS content (mg L^−1^)
	Carbohydrates	Proteins	Lipid content	
**0 (Control)**	76.51 ± 0.61^e^	164.43 ± 2.25^e^	364.4 ± 4.23^e^	1.67 ± 0.22^e^
**10**	70.38 ± 1.05^d^	156.99 ± 2.51^d^	401.0 ± 4.45^d^	33.38 ± 0.08^a^
**25**	60.34 ± 0.19^c^	143.66 ± 3.12^c^	448.3 ± 3.86^c^	26.62 ± 0.36^b^
**50**	52.56 ± 0.36^b^	135.19 ± 3.44^b^	515.7 ± 7.07^b^	13.48 ± 0.08^c^
**100**	42.20 ± 0.66^a^	125.04 ± 2.84^a^	553.1 ± 6.70^a^	3.33 ± 0.22^d^

*Different superscript letters in the same column indicated significant differences (at *P* ≤ 0.05) of each studied parameter compared to the control (cultures without LDPE-MPs treatment).

#### Total carbohydrate and EPS contents

The results showed that the total carbohydrate content of *C. muellerii* cultures was negatively decreased due to LDPE-MPs treatment at 100 and 250µm particle sizes during 6 days of exposure (Table 2). Significant (*p* ≤ 0.05) decrease in the carbohydrate content by 11.99, 33.93, 44.78, and 54.54% less than the control was detected for* C. muellerii* cultures exposed to 10, 25, 50 and 100 mg L^−1^ LDPE-MPs of 100 µm particle size, respectively (Table 2). Likewise, cultures treated with 250 µm particle size displayed a significant decline in the carbohydrate content by 8.01, 21.13, 31.30, and 44.84% at the same previous concentrations, respectively. The lowest carbohydrate content of 34.78 mg g^−1^ DW was estimated when 100 mg L^−1^ LDPE-MPs of 100 µm particle size were added to the cultures. For the EPS content, the results in Table 2 showed a very significant (*p* ≤ 0.05) increase in the culture content by 12.75, 8.84, 5.76, and 1.99 folds than that estimated for the control cultures at 10, 25, 50 and 100 mg L^−1^ LDPE-MPs of 100 µm particle size, respectively (Table 2). A higher increase by 19.98, 15.94, 8.06, and 5.45 folds was also estimated in cultures exposed to the same concentrations of LDPE-MPs with100 µm particle size, respectively. The lowest EPS content of 3.33 mg L^−1^ was estimated when 100 mg L^−1^ LDPE-MPs of 100 µm particle size were added to the cultures.

#### Total lipid content

On the contrary to the total protein and carbohydrate contents, the results revealed a significant (*p* ≤ 0.05) stimulatory effect on the lipid content of *C. muellerii* after 6 days of exposure at all tested concentrations of LDPE-MPs compared to the control cultures (Table 2). The lipid content was increased in relation to the LDPE-MPs’ concentrations in the medium. At LDPE-MPs of 100 µm in size, a significant lipid accumulation by 5.76, 17.62, 32.90, and 47.78% was evaluated over the control cultures at the doses of 10, 25, 50 and 100 mg L^−1^, respectively (Table 2). Equally, cultures containing 250µm of LDPE-MPs resulted in a significant enhancement in the lipid content by 10.04, 23.02, 41,52, and 51.78% at the same polymer concentrations, respectively. The highest lipid content of 553.1 mg g^−1^ DW was got at a 100 mg L^−1^ dose and 250 µm particle size of LDPE-MPs exposure (Table 2).

### Effect of LDPE-MPs treatment on the antioxidant enzymes (Oxidative stress) of* C. muellerii* cultures

#### CAT activity

**Fig. 7:**
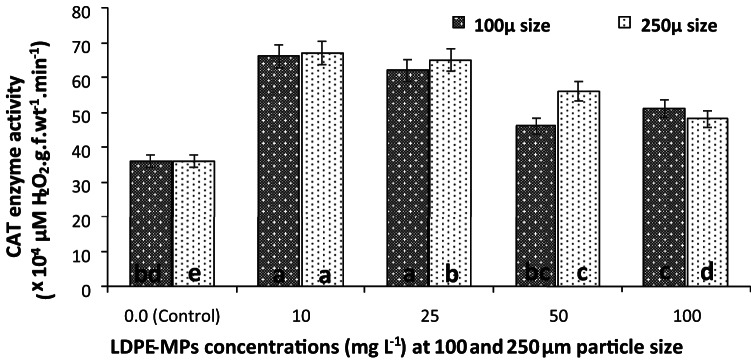
CAT activity of *C. muellerii* cultures under different concentrations of LDPE-MPs (mg L^−1^) measured after 6 days of exposure with particle sizes of 100 and 250 µm. Error bars showed the SD for three replicates. Columns with different letters indicated significant difference between the polymer concentrations at *P* ≤ 0.05.

The effect of LDPE-MPs treatment on CAT activity of* C. muellerii* cultures was illustrated in Fig. 7. The promoting effect was high at the lower concentrations of the polymer, then decreased at the higher ones. Cultures treated with 100 µm of LDPE-MPs promoted significantly the CAT activity by 83.33, 72.22, and 41.67% at 10, 25 and 100 mg L^−1^ of LDPE-MPs, respectively, over the control. However, an insignificant (*p* ≤ 0.05) increase in CAT activity was recorded in the culture treated with 50 mg L^−1^ LDPE-MPs (Fig. 7). Likewise, a significant increase in the CAT activity of *C. muellerii* cultures by 86.11, 80.56, 55.56, and 33.33% was recorded when using a 250 µm particle size of LDPE-MPs at the same tested concentrations, respectively. The maximum CAT activity (67 × 10^–4^ µM H_2_O_2_.g.f.wt^−1^.min^−1^) of* C. muellerii* cultures was recorded at LDPE-MPs of 10 mg L^−1^ dose and 250 µm particle size treatment.

#### SOD activity

**Fig. 8:**
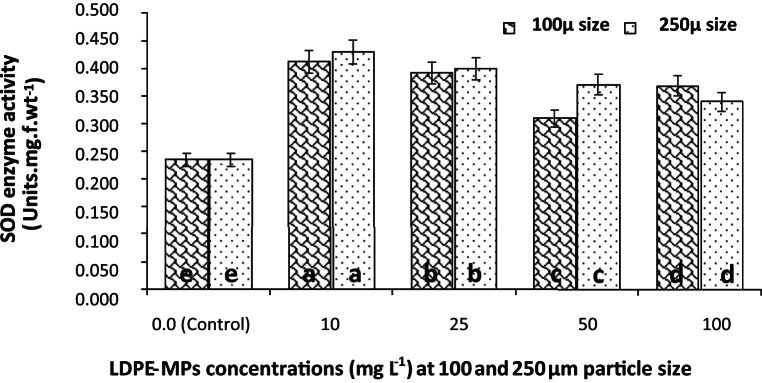
SOD activity of *C. muellerii* cultures under different concentrations of LDPE-MPs (mg L^−1^) measured after 6 days of exposure with particle sizes of 100 and 250 µm. Error bars showed the SD for three replicates. Columns with different letters indicated significant difference between the polymer concentrations at *P* ≤ 0.05.

The influence of LDPE-MPs treatment on the SOD activity of *C. muellerii* cultures was shown in Fig. 8 after 6 days of exposure. The promoting effect was higher at the lower concentrations of the polymer and then decreased at the higher ones. The treatment of cultures with 100 µm particle size at 10, 25, 50 and 100 mg L^−1^ of LDPE-MPs exhibited a significant (*p* ≤ 0.05) promoting effect on SOD activity by 75.64, 67.09, 32.05, and 57.26%, respectively, over the control (Fig. 8). Similarly, a significant enhancement in the SOD activity by 83.33, 70.51, 58.55, and 44.87%, respectively was recorded in cultures exposed to 250 µm of LDPE-MPs at the same concentrations, respectively. The highest estimated activity of SOD was 0.429 units. mg. f. wt^−1^ obtained at LDPE-MPs treatment of 10 mg L^−1^ dose and 250 µm particle size.

### Evaluating the mechanism of interaction between *C. muellerii* cells and LDPE-MPs

#### Detection of the adsorbed LDPE-MPs on *C. muellerii* cells by the microscopic examination

According to the abovementioned results, the highest estimated quantity of LDPE-MPs was achieved in *C. muellerii* cultures treated with a 100 mg L^−1^ dose and 100 µm particle size. Therefore, these cultures were used for the microscopic examination at the end of the incubation period (6 days). The optical microscopic photos of *C. muellerii* control cultures (cultures grown without polymer addition) showed homogenously distributed cells in the cultures with regular shapes and intact frustules of silicified cell walls. The frustules contain four long and thin spines (setae) that extend from the corners, which connect the frustules or multiple cells together, creating a colony of cells (as previously shown in Fig. 3).

**Fig. 9:**
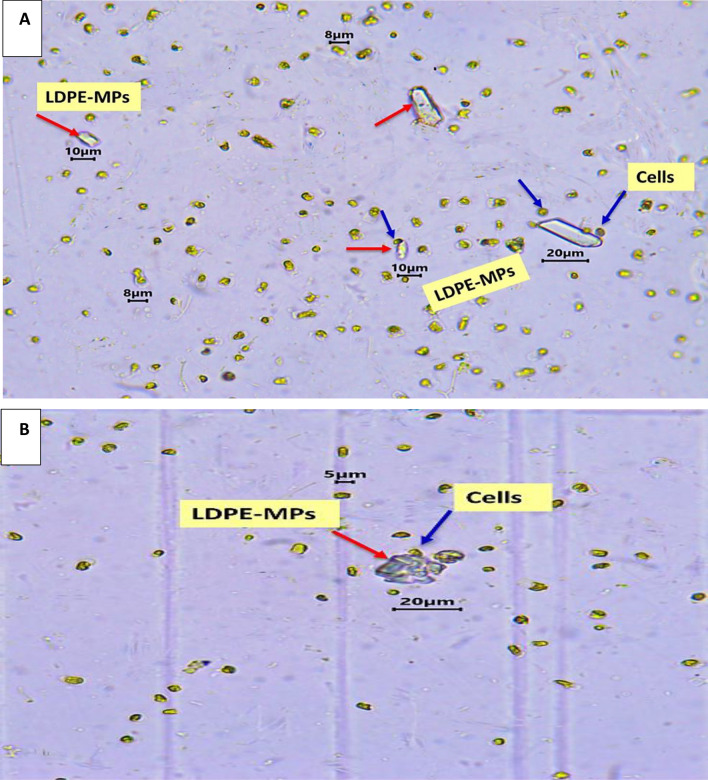
The optical microscopic Figure of *C. muellerii* cells treated with LDPE-MPs of 100 µm particle size (**A** and **B**). Note the formation of hetero-aggregate clusters. Red arrows point to MPs particles around the cell and blue arrows point to *C. muellerii* cell.

The morphological characteristics of LDPE-MP fragments, including shape, size, and color were also observed by optical microscopy. It showed white, irregular plastic particles with a size range from 1 to 250 µm in diameter (as previously shown in Fig. 1). Furthermore, the optical microscopic photos showed morphological changes on the *C. muellerii*’ cell surfaces, damage of the frustules’ cell walls, and loss of the viability under LDPE-MPs treatment (Fig. 9), compared to the control cells grown without polymer addition. Additionally, *C. muellerii* cells were clearly wrapped by LDPE-MPs treatment. And hetero-aggregate clusters were formed between LDPE-MPs and *C. muellerii* cells, which were confirmed by the results of the SEM images.

**Fig. 10:**
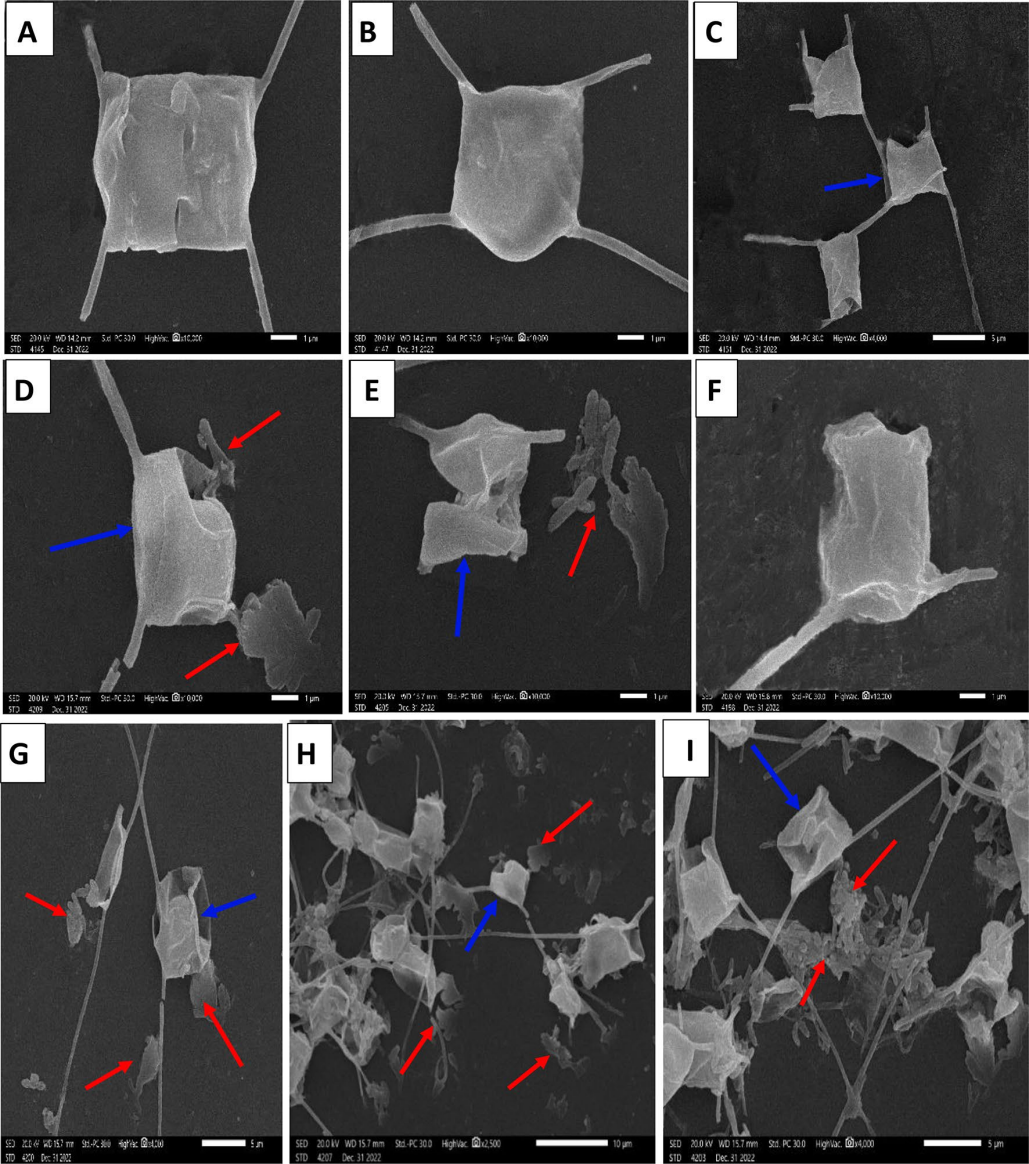
SEM images showing the interaction between LDPE-MPs of 100 μm particle size and* C. muellerii* cells after 6 days of exposure. Images A, B: single cells of *C. muellerii* in control cultures without LDPE-MPs (× 10.000); C: chain of* C. muellerii* cells in control cultures (× 10.000); D-F: physical damages caused by LDPE-MPs treatment on *C. muellerii* cell frustules (× 10.000); G and H: LDPE-MPs adsorbed on the diatom cells of *C. muellerii* and forming hetero-aggregates (× 4.000, × 2.500, respectively); and image I (× 4.000) showed homo-aggregates of LDPE-MPs particles. Red arrows point to MPs particles around the cell and blue arrows point to *C. muellerii* cell.

In the same direction, SEM analysis was used to observe changes in the surface morphology of *C. muellerii* cells after exposure to LDPE-MPs. No visible changes were apparent for *C. muellerii* control cells. They appeared with a regular shape of intact frustules, a smooth cell surface, and a size of about 2–4 μm as shown in Fig. 10 A-C. However, *C. muellerii* cells treated with LDPE-MPs showed evident changes. Numerous particles of LDPE-MPs were clearly observed aggregating or adhering outside the cell surfaces of *C. muellerii*, forming diatom-MP heteroaggregate structures (Fig. 10G, H; red arrows).

The SEM images also showed extracellular polymeric substance (EPS) excreted from the cells to the medium. These substances seemed to have a specific aggregation effect on the LDPE-MP particles and facilitate their adsorption on the surface of the diatom cells. The heteroaggregates were consisted of LDPE-MP fragments trapped in the EPS matrix outside* C. muellerii* cells (Fig. 10G, H). Homoaggregates between LDPE-MP particles were also molded by the EPS matrix (Fig.  10; I). Furthermore, the SEM images displayed physical and structural damage to *C. muellerii* cell surfaces due to LDPE-MP treatments. The frustules’ cell walls were destructed, cracked, and wrinkled with the appearance of cavitations and pits on the cell walls, as shown in Fig. 10D–F. The spines or setae of the cells were also broken, detached, and intermingled with the MPs heteroaggregates, which induced growth inhibition of the *C. muellerii* cultures*.*

#### FTIR analysis of *C. muellerii* cells treated with LDPE-MPs

The results of FTIR analysis of *C. muellerii* cultures treated with LDPE-MPs of 100 and 250 µm particle sizes at different concentrations showed the same peaks for the control cultures. However, there were changes in the percentage of transmittance (%T) for some peaks, implying changes in the intensity of their functional groups (i.e., components), compared to the control ones (Fig. 7S and 8S). The treatment of* C. muellerii* cultures with LDPE-MP of 100 µm particle size at 10 and 100 mg L^−1^ doses, resulted in the following changes compared to the control peaks (Table 3). A decrease in the intensity of the strong, broad peak at a wavelength of 3308 cm^−1^ represented the amine (N–H) and hydroxyl (O–H) groups of the protein and polysaccharide components. A decrease in the intensity of peaks at the wavelengths 2929 and 1657 cm^−1^ indicated the alkane (C-H) and carbonyl (C = O) stretching of the protein components, respectively. A decrease in the intensity of the peak at 1536 cm^−1^ indicated the aromatic nitro compounds (N–O) at 10 mg L^−1^, which disappeared at 100 mg L^−1^. On the contrary, an increase in the intensity of the weak, sharp band at 1449 cm^−1^ revealed the methyl group (CH_3_-) of the protein component at 100 mg L^−1^. New appearances of carboxylic acids’ carbonyl group (C–O–C), which noticed at 1233 cm^−1^ at 100 mg L^−1^. A decrease in the intensity of a strong, sharp peak at 1080 cm^−1^ revealed the presence of carbonyl of carboxylic acids (C–O–C) and phosphate (P = O) groups in polysaccharides. Moreover, an increase in the intensity of weak, sharp, and broad bands at a wavelength range of 790–855 cm^−1^ represented the presence of alkyl groups (C–H and CH_2_) in the polysaccharide and protein components at 100 mg L^−1^ (Table 3, Fig. 7S).

**Table 3:** FTIR analysis showing the distribution of functional-group bands of *C. muellerii* cells under treatment with LDPE-MPs of 100 and 250 µm particle sizes at concentrations of 10 and 100 mg L^−1^ after 6 days of exposure. Control cultures were without addition of LDPE-MPs.

Functional group (bond type)	Compound class	Absorption mode of bands	Frequency range (cm ^−1^)	Control culture	LDPE-MPs at 100 µm size		LDPE-MPs at 250 µm size	
					10 mg L^−1^	100 mg L^−1^	10 mg L^−1^	100 mg L^−1^
O–H stretching N–H stretching	Hydroxyl- alcohol Amine	strong broad	3550–3200 3500–3400	3308.3	3421.1	3413.3	3403.7	3412.4
C-H stretching	Alkanes	weak sharp	3100–2850	2929.3	2928.4	2924.5	2928.4	2928.4
S–H stretching	(Thiol) Sulfhydryls	weak sharp	2600–2100	2372.9	2372.9	2372	2372.9	2373.9
C = O stretching	Carbonyl	strong sharp	1800–1600	1657.5	1648.8	1648.8	1648.8	1648.8
N–O stretching	Aromatic nitro compounds	strong and weak sharp	1555–1485	1536.9	1551.5	–	1539.9	1540.9
C-H, CH_3_ bending	Methyl	weak sharp	1470–1350	1449.2	1449.2	1470.5	1449.2	1450.2
C–O–C stretching	Carbonyl of carboxylic acids (Alcohol)	weak broad	1300–1000	–	–	1233.3	–	–
C–O–C stretching P = O stretching Si–O-Si stretching	Carbonyl of carboxylic acids Phosphates Organic siloxane or silicone	strong sharp	1300–1000 1130- 1040 1095–1075	1080.9	1072.2	1075.1	1083.8	1081.9
C-H, CH_2_ bending	Alkyl	weak sharp and broad	900–670	790.7	853.3	855.3	704.9	852.4
C-S stretching	Disulfides	weak broad	705–570	637.4	622.9	594.9	–	602.7
S–S stretching	Aryl disulfides	weak sharp	500–430	454.2	468.6	443.6	467.7	459.9

In the same manner, the treatment of* C. muellerii* cultures with LDPE-MPs of 250 µm particle size at 10 and 100 mg L^−1^ doses showed the following results compared to the control (Table 3). An increase in the intensity of a strong, broad peak at a wavelength of 3308 cm^−1^ was noticed, indicating the amine (N–H) and hydroxyl (O–H) groups of protein and polysaccharide components at 100 mg L^−1^. A decrease in the intensity of peaks at 2929 and 1657 cm^−1^ was detected, representing the alkane (C-H) and carbonyl (C = O) stretching of the protein components, respectively. A decrease in the intensity of the peak at 1536 cm^−1^, which indicated the presence of aromatic nitro compounds (N–O). On the other side, an increase in the intensity of the weak, sharp band at 1449 cm^−1^ revealed the presence of the methyl group (CH_3_-) of the protein component at 100 mg L^−1^. An increase in the intensity of the strong, sharp peak at 1080 cm^−1^ was indicative of a carbonyl of carboxylic acids (C–O–C) and phosphate (P = O) groups in polysaccharide components. Further, an increase in the intensity of the weak, sharp, and broad bands at a wavelength range of 790–855 cm^−1^ showed the presence of alkyl groups (C–H and CH_2_) in the polysaccharide and protein components at 100 mg L^−1^ (Table 3, Fig. 8S).

#### Detection of the adsorbed LDPE-MPs on *C. muellerii* cells by the DSC and combustion techniques

**Fig. 11:**
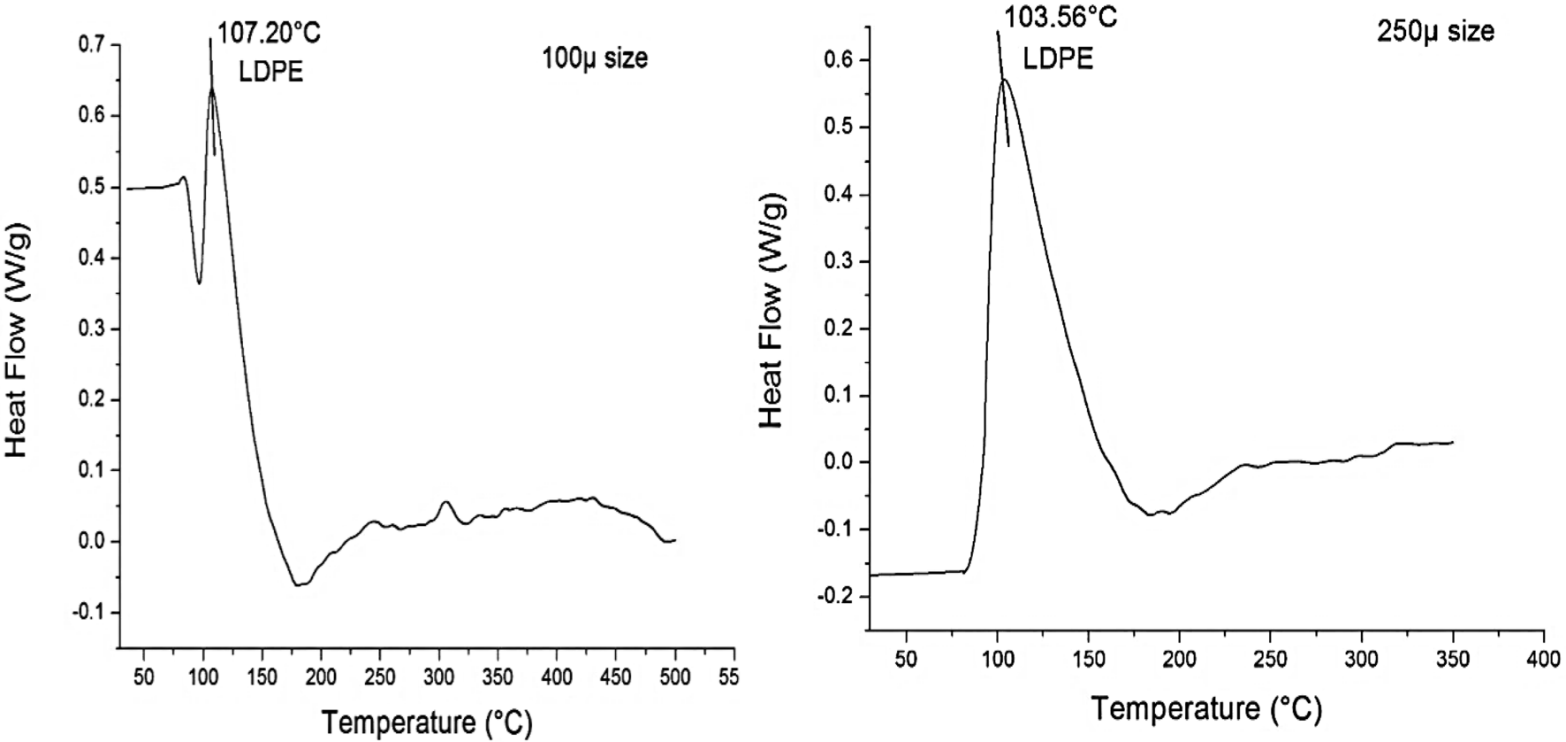
The DSC thermogram for the LDPE-MPs detected on *C. muellerii* cell surfaces from cultures treated with 100 and 250 µm particle sizes, respectively.

The DSC data including the characteristic heat enthalpy, the melting temperature (*Tm*), and area under the melting peak were utilized to identify the accumulated LDPE-MPs on the *C. muellerii* cells (Fig. 11). The characteristic *Tm* of LDPE-MPs was detected at 107 and 103°C, representing the polymers in the cultures treated with 100 and 250µm particle sizes of LDPE-MPs, respectively.

The weight loss using the combustion technique was used to determine the accumulated quantity of LDPE-MPs on the surface of *C. muellerii* cells. The results showed the weight loss (%) as a function of the specific temperature degree for the decomposition of the sample components (dried algal biomass), as shown in Fig. 9S. The lowest weight loss of *C. muellerii* cells was at 150 °C, while the highest weight loss was at 300 and 450 °C due to natural polymers and plastic material decomposition, respectively. The weight loss percentage resulted from LDPE-MPs decomposition was higher in the biomass treated with small particle sizes of LDPE-MPs (100µm) compared to the biomass treated with large particles (250µm) at both examined concentrations of 10 and 100 mg L^−1^. Additionally, the results showed that the weight loss % was greater in *C. muellerii* biomass treated with LDPE-MPs concentration of 100 mg L^−1^ than that treated with the concentration of 10 mg L^−1^ for both particle sizes of the polymer (Fig. 9S).

Based on the thermal analysis results, the weight of LDPE-MPs accumulated on the cell surface of *C. muellerii* was quantitatively calculated (Table 4). The results showed that higher weights of LDPE-MPs were accumulated on the cells treated with 100 mg L^−1^ rather than those treated with 10 mg L^−1^. Besides, the capability of *C. muellerii* cells to accumulate small- sized LDPE-MPs of 100 µm was higher than the larger- sized ones of 250 µm. The highest adsorbed weight of LDPE-MP particles on *C. muellerii* cell surfaces was 0.334 g g^−1^ DW estimated in cultures treated with 100 mg L^−1^ and 100µm particle size of LDPE-MPs. This value represented 33.04% of the initial weight of LDPE-MPs added to the culture, while the residual LDPE-MPs was 66.96% (Table 4).

**Table 4:** LDPE-MP weights accumulated on the cell surfaces of *C. muellerii.*

LDPE-MPs size (µm)	LDPE-MPs concentration (mg L^−1^)	Adsorbed LDPE-MPs (%)	Residual LDPE-MPs (%)	Weight of adsorbed LDPE-MPs (g g^−1^ DW)
**100 µm**	10	29.41	70.59	0.030
	100	33.04	66.96	0.334
**250 µm**	10	11.49	88.51	0.012
	100	16.28	83.72	0.167

## Discussion

As a result of increasing plastic pollution in the world’s oceans, potential diverse effects on microalgae, including phytoplankton, were recently raised^48^. The exposure of algae and other microorganisms to MPs is the best choice to assess the aquatic toxicity and to detect the environmental risks caused by the MPs’ pollution in aquatic environments^49^.

In the present study, the growth curve of* C. muellerii* indicated three successive phases of growth: the exponential phase followed by the stationary phase and a long declining phase, while the lag phase was not observed. Shevchenko et al*.*^50^ studied the growth curve of *C. socialis* in F/2 medium and obtained four stages of growth: a short lag phase for one day, four days of exponential phase, and a very short stationary stage on the 7^th^ day with a growth rate of 0.3 divisions per day. The same study^50^ reported a maximum growth rate at the beginning of the exponential phase, which was in line with the current results because the highest cell density of* C. muellerii* was estimated at the late exponential growth phase.

### Influence of LDPE-MPs exposure on *C. muellerii* growth

Most of the published research has assessed the effects of MPs on green microalgae, but data about the effects of MPs on other phytoplankton groups, especially diatoms, is still insufficient compared to their bulk ratio in the phytoplankton community. Concerning the influence of LDPE-MPs on the growth of *C. muellerii* as cell density and OD_680_ value, the present study indicated inhibition of the growth at all tested concentrations of LDPE-MP compared to the control. The inhibition effect was dependent on the concentration, size, and exposure time to MPs. This was also confirmed by the specific growth rate (d^−1^) results. Previous studies showed that different types of MPs can influence the growth of microalgal species, causing direct physical damage on the algal cells, thereby inhibiting their growth and reflecting on aquatic food webs^51^. Growth inhibition of 57% on the marine microalgae *Dunaliella tertiolecta* was reported after 72 h exposure to uncharged PS particles of 0.05 µm size and 250 mg L^−1^ concentration^52^. Likewise, Zhang et al.^49^ showed a negative effect of 1 µm mPVC on the growth of *Skeletonema costatum* diatom, with a maximum inhibition percentage of 39.7% at a high concentration (50 mg L^−1^) of the MPs. In addition, Song et al*.*^53^ reported an obvious inhibition effect when using 74 μm particle size of PP, PET, PE, and PVC on the growth of *Phaeodactylum tricornutum* MASCC-0025 at a concentration of 200 mg L^−1^. A maximum inhibition percentage of 21.1% was estimated after 4 days of cultivation with PVC-MPs. Furthermore, the interaction between *Spirulina* sp*.* cultured with PE and PP-MPs of 0.5–1 mm particle size was studied at a concentration of 500 mg/500 mL culture volume^54^. A significant decrease in the growth rate of *Spirulina* sp*.* culture was recorded at 0.0228 day^−1^ and 0.0221 day^−1^ for PE and PP treatments, respectively, compared to the control rate of 0.0312 day^−1^.

In contrast to the study finding, some studies reported no significant impact of MPs exposure on the growth of microalgae. For instance, A study investigating virgin LDPE microbeads found no acute growth inhibition in the diatom *P. tricornutum* at environmentally relevant concentrations up to 1.25 × 10^3^ particles L^−1^ (0.05 mg L^−1^), though nano-sized plastic particles might have a greater potential for harm due to their ability to penetrate cell membranes^12^. Lagarde et al.^55^ investigated the influence of PP and HDPE on the growth of the green microalga *Chlamydomas reinhardtii*. No obvious impact of both polymers on the microalga growth was observed even at high concentrations of 400 mg L^−1^ and a particle size > 400 µm. In addition, Prata et al.^56^ inspected the influences of MPs of a size range from 1 to 5 μm on the specific growth rate of the marine green microalga *Tetraselmis chuii*. The results showed no significant effects on the growth rate after 96 h of exposure and up to 41.5 mg L^−1^ concentration. In the environment, exposure to MPs’ pollution is mainly related to lower trophic levels of the food webs, including phytoplankton^9^. Negative impacts on primary producers may change food resources and translate adverse effects to higher consumers, including humans. For the current study, a pilot estimation of LDPE-MPs concentration in the EH water was assessed to be in the range of 10 to 15 mg L^−1^. Therefore, the investigated concentrations of LDPE-MPs were arranged among and higher than the relevant environmental values in the EH area in order to assess the capability of *C. muellerii* diatom to tolerate a broader range of MPs’ stress concentrations. Furthermore, the two tested particle sizes were compatible with the detected range^31^ (up to 500 μm) of LDPE-MPs in the EH area to validate the results compared to the typical environment. In this context, many previous studies mentioned various factors such as the MP-particle type, size, surface properties, concentration, and exposure duration, which can affect the toxicity of MPs on the microalgae cells^55^. Different plastic types may alter the adsorption capacities of microalgae and thus affect their toxicity^57^. Besides, the type of microalgal species, its shape, size, and cell wall structure may influence the adsorption of MPs on the microalgae cells^49^.

Many other studies, have formerly adopted that the adsorption of MPs on the surface of microalgal cells harmed their growth^32,58^. The adverse effects include cell wall damage, interference with nutrient uptake from the medium, restriction of light energy and essential-element transmission, interference with CO_2_/O_2_ exchange between the cells and the culture medium, and/or decreasing cell mobility^58,59^. Moreover, the harmful metabolites of the microalgae could be preserved inside the cell by the action of hetero-aggregates formation, causing disturbance of the algal growth^60,61^. In this connection, the results of the current study are in the same line with the aforementioned reports. Moreover, the potential release or leaching of toxic chemical additives such as plasticizers and heavy metals, incorporated during the manufacturing process of MPs, could be another reason for explaining the indirect toxicity of MPs to algae^53^^,^^62^. It could interfere with the biological processes and decrease the metabolic activity of the cells^62^. It should be noted that, according to the current results, the inhibition of growth rate was significantly reduced for both LDPE-MPs particle sizes at all tested concentrations on the 6^th^ day of the experiment. This may be due to the ability of *C. muellerii* cells to adapt to MPs-induced stress, as indicated by the enhanced growth rate and physiological state. Many studies reported that algal biomass, photosynthesis, and cell structure could recover after MP exposure during growth from the late log phase to the stationary phase^57^^,^^63^.

The results showed a reduction effect on the content of photosynthetic pigments, chlorophyll *a, c,* and carotenoid, which was dependent on the experimental concentration, particle size, and time of exposure to LDPE-MPs. The more pronounced toxic effect on the carotenoid than the chlorophyll content of *C. muellerii* cells denotes the shield role of carotenoids for the protection of chlorophyll against the photooxidation effect in the presence of LDPE-MPs. The reduction in the pigment content might be attributed to two reasons: firstly, the presence of MPs in the culture could induce accumulation of intracellular ROS, causing damage to the cell structure and blocking chlorophyll synthesis. Secondly, because MPs have a large surface area and strong adsorption ability, heteroaggregates between *C. muellerii* cells and MP particles were easily formed, and so reduced the microalgal activity^55,60^. In addition, MPs could interrupt the photosynthetic electron transport, which resulted in mitigating O_2_ evolution by the PSII reaction center^64^. The presented results were consistent with the earlier studies reporting that exposure to external environmental stress, including MPs, could decrease the actual photosynthetic efficiency of some algae^65^. A decrease of the total chlorophyll content in the dinoflagellate *Karenia mikimotoi* cells was reported after 96 h of exposure to PVC-MPs of 1 µm in size^34^. Similarly, Zhang et al.^49^ found that the total chlorophyll content and the photosynthetic efficiency of *S. costatum* cells were decreased by 20% and 32%, respectively, under mPVC treatment (1 µm in size, 50 mg L^−1^ in concentration). Contrary to the presented findings, Long et al.^60^ observed no adverse effects of the uncharged PS-MPs (2 µm and 0.04 µg mL^−1^) on the chlorophyll content of *C. neogracile* cells. Likewise, Song et al. ^53^ reported no significant difference in the chlorophyll and carotenoid contents between the control and the treated algal cultures of* Chlorella* sp*.* L38 due to MPs exposure. While a gradual increase in the chlorophyll concentration of *P. tricornutum* MASCC-0025 cultures was obtained after PE and PET- MPs were added.

Regarding the size effect of LDPE-MPs, the results implying that the toxic effect of LDPE-MPs on the growth and photosynthetic pigments of* C. muellerii* cells was oppositely related to the LDPE-MPs’ size (i.e., toxicity increased with decreasing particle size). Numerous studies reported that the particle size was one of the important factors for determining the toxicity mechanism and negative impact of MPs on microalgae^66,67^. Sjollema et al.^52^, who examined the effect of PS particles of different sizes on the growth of the microalgae *D. tertiolecta* for 72 h. The small PS-MP beads of 0.05 µm caused a reduction in the culture light intensity of up to 34% while inhibiting the algal growth by 45% compared to the control cultures at the highest concentration of 250 mg L⁻^1^. However, the larger PS beads of 0.5 and 6 µm caused a reduction of *D. tertiolecta* growth rate by 11% and 13% for both bead sizes, respectively. Still, no significant inhibition effect on the photosynthesis was observed, implying that the shading effect was not the only reason for the toxic effects of MPs. Furthermore, the toxic effects of LDPE-MPs exposure on the growth of *C. muellerii* may be explained on the basis that large-sized MP particles can float on the surface of the culture medium, causing serious effects by blocking light energy and nutrient transport, as well as inducing shade effects that hinder photosynthesis process and inhibit growth^32,53^. The negative effect of large particles may be trivial compared to the small-sized MPs because light can pass through the transparent wall of the flask into the medium and satisfy the needs of algal growth^49^. In fact, small-sized MPs, of large surface area, can enter the culture medium and interact with the microalgal cell membranes through adsorption and aggregation. This large surface area can also induce shading, block light entrance, and prevent gas exchanges, thus causing more oxidative stress to the algal cells^67^. In this respect, Chen et al.^68^ revealed that PS- NPs could be taken and absorbed into microalgae cells through pores of the cell wall more than PS-MPs. It could cause a significant reduction in the density of microalgae and their photosynthesis capacity, which supported that the cellular internalization of plastic debris into microalgal cells was dependent on its size. These findings align with the results of the present study, as the growth indices in *C. muellerii* were more significantly reduced when exposed to 100 µm particle size of LDPE-MPs compared to those exposed to 250 µm, where the shading effect may have been more pronounced.

### Effect of LDPE-MPs exposure on the biochemical compositions of *C. muellerii* cells

The inhibition percentage of the protein and carbohydrate contents was directly proportional to the LDPE-MP concentrations in the medium. The results showed that the proximate content of *C. muellerii* cultures exposed to 250 µm LDPE-MP was higher than that of 100 µm at the same concentrations, implying that the large particle size of LDPE-MPs caused higher productivity of biochemical compositions than the smaller ones.

Extracellular polymeric materials are polymers that can be actively produced during the metabolism of the microalgal cells. They can be released from the cells in response to conditional stress as a defense mechanism and to prevent the penetration of foreign substances through the cell surface^69^. The main components of these materials include exopolysaccharides (EPS), proteins (enzymes and structural proteins), nucleic acids, and lipids^70^. In the current study, the decrease in the protein and carbohydrate contents of *C. muellerii* cells could be attributed to the toxic effect of LDPE-MPs on the photosynthetic pigments, which directly affected the photosynthetic activity and their ability to synthesize macromolecules. The reduction in these proximate contents could have serious ecological implications on the functioning of the food web by decreasing the nutritional quality of microalgae as primary consumers^71^. In close agreement with the current finding, Hadiyanto et al.^54^ reported a decrease in the protein content of *Spirulina* sp*.* following PE and PP-MPs exposure. The protein content decreased by 0.121% and 0.108% of the dry biomass with PE and PP treatments, respectively. The EPS production by *Spirulina* sp*.* cells was more increased after PE-MPs treatment than that observed after PP-MPs treatment^54^. However, Lagarde et al.^55^ investigated the interactions between *C. reinhardtii* cells with HDPE and PP-MPs in the cultures for 70 days of contact. Their results showed over-expression of *C. reinhardtii’s* genes involved in the sugar biosynthesis pathways for both treatments compared to the control cultures. Higher EPS production was observed with HDPE cultures than those treated with PP ones, implying that the overexpression of genes might be related to the higher EPS excretion by the treated cells. Another study by Song et al.^53^ found that exposure of *Chlorella* sp*.* L38 and *P. tricornutum* MASCC-0025 to MPs could stimulate EPS generation as a protection defense by 1.4 and 2.2 times in both microalgae cultures, respectively, over the control cultures. In this connection, the results of the current study showed secretion of high quantities of EPS in response to LDPE-MPs treatments, which was proportional to the lower concentration and larger size of the MPs. This facilitates the formation of homo- and heteroaggregates between the diatom cells and MPs^8,55^.

Considering, the lipid content of *C. muellerii* cells, the results displayed a significant (*p* ≤ 0.05) stimulating effect at all tested concentrations and sizes compared to the control cells. These findings were comparable with those of Wang et al.^72^, who studied the effects of various MP types and sizes on the lipid production of *Scenedesmus quadricauda*. The treatment with PP-MPs of 50 µm size at a concentration of 250 mg L^−1^ resulted in a substantial accumulation of neutral lipids (65.40%). The observed increase in the lipid content could be attributed to the interaction between LDPE-MPs, as a stress factor, and *C. muellerii* cells during the culture period. Lipids accumulate as a cell response by modulating its energy metabolism to properly overcome the stress conditions caused by MP exposure and to maintain normal growth, photosynthesis, and membrane integrity^71^. As formerly reported, exposing microalgae to MPs significantly up-regulates the KCS genes associated with long-chain fatty acid synthesis^62^. In laboratory cultures, microalgae generally tend to accumulate neutral lipids, mainly triacylglycerol, in specific organelles called lipid bodies during the stationary growth phase. This could happen upon stresses such as nutrient limitation, elevated temperatures, unfavorable light intensities, alkaline pH, high salinity, or dehydration^73^. Thus, microalgal oil bodies could serve as an energy source for maintaining a healthy cellular status. In contrast to the study finding, Seoane et al.^71^ investigated the toxicity effects of PS-MP beads of 0.5 and 2 µm sizes at 2.5 µg mL^−1^ on the esterase activity and neutral lipid content of the marine diatom *C. neogracile*. A significant decrease in the cellular esterase activity and neutral lipid content was recorded after 24 h of MP exposure compared to the control cells.

In photosynthetic microorganisms, including microalgae, antioxidant enzymes such as SOD and CAT can be used as endogenous protective mechanisms against unbalanced reactive oxygen species (ROS) cellular production^74^. Many researchers have assayed the activity of these enzymes to evaluate the stress status and toxicity of pollutants^75^^,^^76^. In this context, results of the present study showed a significant (*p* ≤ 0.05) promoting effect on both SOD and CAT activities of *C. muellerii* cells exposed to LDPE-MPs at different concentrations and sizes. The subsequent increase in CAT and SOD activities after LDPE-MPs exposure suggested that *C. muellerii* cells were under oxidative stress. The CAT enzyme catalyzes the decomposition of H_2_O_2_ into H_2_O and O_2_, maintaining a constant H_2_O_2_ level within cells^77^. Accordingly, increased CAT activity in *C. muellerii* cells may be attributable to the activation of H_2_O_2_ generation following LDPE-MPs exposure. Likewise, higher SOD activity in the diatom cells could be linked to the overproduction of superoxide radicals (O_2_^−^) because of LDPE-MP exposure. SOD represents the first line of defense against ROS damage by removing or scavenging of O_2_^–^ to produce non-toxic oxygen and less toxic H_2_O_2_, thus reducing the potential toxicity of pollutants for microorganisms^78^. The enhanced activity of CAT over SOD in *C. muellerii* cells could be owing to a larger induction of H_2_O_2_ than O_2_- upon LDPE-MPs exposure.

However, the particle size of LDPE-MPs did not affect the CAT and SOD enzyme activities at all tested concentrations. These results were consistent with a former study by Zhang et al.^79^, who investigated the acute and chronic toxic effects of Bisphenol A on *Chlorella pyrenoidosa* and *Scenedesmus obliquus* cells. They reported that SOD and CAT activities of both algae were stimulated in all the culture treatments exposed to Bisphenol A compared to the control cultures. As reported by Liu et al.^80^, the increase in SOD and CAT activities confirmed that the addition of LDPE-MPs could stimulate a rapid increase of ROS, including O_2_^−^, hydroxyl radical (·OH) and singlet oxygen (1O_2_), thus causing oxidative damage to the microalgae cells. Liu et al.^80^ found that *S. obliquus* cells produced a higher amount of ROS when the cultures were treated with MPs of 0.1 and 0.5 μm particle size at 50 mg L^−1^, compared to the control cultures without MPs. Also, the positively charged MP types generated more ROS than the negatively charged ones. Similarly, Xiao et al.^81^, found that the SOD activity was markedly increased in cells of the freshwater microalga *Euglena gracilis* after treatment with two sizes (0.1 and 5 mm) of PS-MPs. The SOD activity recorded 22.03 ± 1.79 and 24.59 ± 2.39 U mg^−1^ protein, respectively, compared to 16.38 ± 0.57 U mg^−1^ protein in the control cells, indicating oxidative stress induction in *E. gracilis* cells. In contrast to these finding, Seoane et al.^71^ evaluated the toxicity effects of PS beads of two sizes (0.5 and 2 µm) at a concentration of 2.5 µg mL^−1^ on ROS levels of the marine diatom *C. neogracile* for 72 h without the detection of oxidative stress enzymes in the PS-exposed cells.

### Evaluation of the interaction mechanism between *C. muellerii* cells and LDPE-MPs

In the presented study, optical and SEM images revealed numerous LDPE-MPs aggregating or adhering outside the cell surface of *C. muellerii* in the treated cultures, forming heteroaggregates as well as homo- or self-aggregates in the culture medium. These results combined well with the remarkable increase in the secretion of EPS that was estimated due to LDPE-MPs treatments compared to the control cultures (Table 2). This interaction was promoted by the physiochemical properties of LDPE-MPs as being long chains of ethylene monomers of low density, low crystallinity, and hydrophobicity^12^. The developed aggregates were attached to the diatom cells and triggered physical damage and cytotoxicity, especially at higher doses of the polymer.

These findings were in line with a number of other investigations that shown that microalgae exposed to MP particles adhered to the cell surface, forming hetero-aggregate clusters^82,83^.

Sticky EPS attached to MPs by hydrogen bonding or electrostatic interactions could promote the formation of homo- or heteroaggregation between microalgae cells and/or MPs^62^. The genus *Chaetoceros,* in particular, is well known to produce large amounts of transparent EPS^84,85^. As reported by Nolte et al.^74^, adsorption of MPs to the microalgal cell surface is dependent on the physicochemical properties of the plastic, such as particle size and particle surface charge, and the morphological characteristics of the algae. Lagarde et al.^55^ used SEM images to confirm the interaction and formation of heteroaggregates between PP and HDPE-MPs with the green microalga *C. reinhardtii* microalga. Likewise, SEM results conducted by Mao et al.^57^ revealed that PS-MPs of 100 mg L^−1^ dose, and different sizes could adhere to the surface of *C. pyrenoidosa* cells after 30 days of exposure, forming heteroaggregates.

It is difficult to characterize MPs using a single detection method, owing to the differences in size, structure, and type of the polymer^86^. FTIR spectra is an effective and non-destructive technique for identifying MP polymer composition and detecting organics functional groups of the algal biomass with high accuracy results^87^. In the present study, the FTIR results proved that exposure of *C. muellerii* cells to LDPE-MPs could reduce the quality of *C. muellerii* biomass by decreasing the protein and polysaccharide contents and even the disappearance of their representative functional groups compared to the control cells. These observations also supported the findings that *C. muellerii* cells were more damaged by the smaller-sized LDPE-MPs, which bind to the cells by adsorption causing cell damage and reduce biomass quality. These results were in match with the explanations of Hadiyanto et al.^54^, who studied the FTIR spectra of *Spirulina* sp*.* biomass and found a decrease in the intensity of NH_2_, amide (CO–NH), and C = O groups of the protein components after treatment with PE and PP-MPs. The authors also observed a decreased intensity of the O–H groups of polysaccharides due to PE-MPs addition. Furthermore, The FTIR results of Wang et al.^72^ proved that the addition of MPs reduced the intensity of protein, lipid, and carbohydrate-related bands in the biomass, thus influencing algal cell structure. An explanation of this physical damage was also introduced by Dianratri et al.^88^ that the functional groups such as COO- and OH- from protein and polysaccharides have negative ions (anions), whereas the heavy metals in MP particles have positive ions (cations), allowing them to bind by biosorption. This bonding may cause damage to *C. muellerii* cells and decreases the biomass quality, especially the protein and polysaccharide contents. This explanation is in consistent with the early study by Clement et al.^89^, who investigated the interaction between titanium oxide (TiO_2_), as an additive in MPs and *Chlorella* sp. and *Scenedesmus* sp. cells. They observed changes in the peak’s intensity of the C = O, NH_2_, OH-, and COO- groups after TiO_2_ interaction. Similarly, the interactions between *Spirulina* sp*.* and the copper (Cu) heavy metal, as another additive in MPs, were found to modify the peaks of the NH_2_, CO–NH, and COO- groups at the wavelength range 1300–1000 cm^−1^. This modification indicated the presence of OH groups, which interacted with Cu ions as one of the main binding sites on the biomass surface^90^.

The thermal analysis techniques were used to facilitate the identification of the chemical composition of MPs where temperature is applied simultaneously to an unknown sample and a reference material^91^. In the present study, the DSC results indicated a strong *Tm* peak represented the characteristic melting points of LDPE at 100 and 250 µm size treatment, respectively of the diatom biomass. The detected LDPE-MPs was accumulated on the diatom biomass, found in seawater, and in sediments in the EH bay as reported by Shabaka et al*.*^31^. The shift in *Tm* values of the same polymer could be attributed to the polymer’s aging, which resulted in the reduction in its crystallinity and melting point. Moreover, the characteristic *Tm* could be affected by the degree of polymer branching, impurities or additives, and its particle size^92^. The TGA using the combustion technique was employed for quantifying the MP materials by measuring the weight loss (as a percentage) of the sample at a particular temperature^93^. In a recent work, Shabaka et al.^46^ successfully applied the combustion technique together with the DSC analysis, for the first time, to quantify MPs contamination in the fish digestive tracts prevailing in the EH area in Egypt. Therefore, this technique was applied for quantifying and monitoring the formation of heteroaggregates between LDPE-MPs and *C. muellerii* cells. The results indicated that the lowest weight loss percentage of *C. muellerii* was at 150 °C, while the highest weight loss was at 300 °C and 450 °C, implying the decomposition of natural polymers and plastic materials in the diatom sample, respectively. This confirmed the thermal decomposition of the adsorbed LDPE-MPs on *C. muellerii* biomass and verified that the LDPE-MPs entrapped in the heteroaggregates could be quantified. In this context, Lagarde et al.^55^ investigated the interactions between PP-MPs and the freshwater microalga *C. reinhardtii*, after 20 days of contact. The authors reported formation of ovoid heteroaggregates between the microalgal cells and PP-MPs of 4.5 mm in size, 2.00 ± 0.48 mm in length, and 1.49 ± 0.55 mm in width at the end of the experiments. The heteroaggregates were composed of 52% PP-MPs and 4% biomass. In addition, the recovered plastic fragments were determined by the weight loss to equal 94.5%, while the 5.5% loss was probably due to the adsorption of PP-MPs trapped into the heteroaggregates. These results were in consistent with the results of the current study, since the largest weight of the adsorbed LDPE-MPs was 0.334 g g^−1^ DW at 100 mg L^−1^ and 100 µm size, which represented 66.96% of the recovered fragments and 33.04% of the adsorbed ones.

It should be mentioned that the enthalpy changes cannot be detected by TGA^91^, but they are obtainable by the DSC measurements. Therefore, a combination of DSC and TGA was suggested for the best detection of MPs. In the current work, the coupling of the two techniques provided useful information on the polymer type, percentage composition, and its quantification (w/w). In addition, these results may provide more important information regarding the potential impact of MPs on the primary producer in the aquatic ecosystems and contribute to further understanding of the mechanism of MP’s toxicity on marine organisms.

## Conclusion

LDPE-MPs exposure at two different particle sizes had significant inhibitory effects on the growth rate, photosynthetic pigments, protein, and carbohydrate contents of *C. muellerii* cells. The toxic effects of LDPE-MPs treatment on the marine diatom were proportionally dependent on the concentration and exposure time to the MPs, while inversely dependent on the particle size of the polymer. The cells of* C. muellerii* developed a defense mechanism to overcome the noxious effects of LDPE-MPs by stimulating lipid accumulation, EPS excretion, and antioxidant enzyme activation. In addition, the exposure of *C. muellerii* cells to LDPE-MPs reduced the quality of the biomass by changing the content of organic functional groups of the protein and polysaccharide compounds. The SEM images revealed that the interaction between *C. muellerii* cells and LDPE-MPs triggered the formation of homo- and heteroaggregates with the aid of the excreted EPS in the medium. The heteroaggregates may be partially responsible for the direct physical and structural damage of LDPE-MPs on the diatom cells. The formation of heteroaggregates seems an adaptive dynamic by the cells to gather LDPE-MPs from the medium and combat their cytotoxicity. The FTIR and heat enthalpy data suggest that homo- and heteroaggregates may form through chemical adsorption interactions. These interactions occur between the active groups on the diatom cells, their ionic charged EPS, and LDPE-MPs. The DSC and combustion techniques were applied for quantifying and monitoring the formation of heteroaggregates between LDPE-MPs and *C. muellerii* cells. To our knowledge, this is the first work to couple both techniques and prove their efficiency for the quantification of the accumulated LDPE-MPs on the algal cell surface. *C. muellerii* cells showed valued capability to accumulate small-sized LDPE-MPs from the cultures, especially at high concentrations of the polymer.

As microplastic contamination becomes a serious danger to the sustainability of healthy aquatic ecosystems, this study introduces a new management approach and alternative eco-friendly solutions to detect and monitor the algal response to plastic exposure. Also, to explore how these microorganisms, as essential primary producers, can adapt to and combat the toxicity of MPs which may be directed to the bioremediation approaches of aquatic ecosystems. Further investigation should continue on other types of phytoplankton species to understand their mechanism of action to withstand the threats of MPs’ pollution. This will be positively reflected in the sustainability of aquatic food webs and their habitats.

## Supplementary Information

Supplementary Information.

## Data Availability

The datasets collected and/or analyzed during the current study are available from the corresponding author on reasonable request.
