# Supplementary material for: The toxicological effects of low-density polyethylene microplastic particles (LDPE-MPs) on the growth and metabolic activities of the marine diatom Chaetoceros muellerii
Source: Sci Rep. 2025 Nov 29;15:42913. doi: 10.1038/s41598-025-27440-9 (PMC12672581; doi:10.1038/s41598-025-27440-9)
Supplement: Supplementary file 1 — Supplementary Information. [file 41598_2025_27440_MOESM1_ESM.pdf]

Supplementary Material for the research article:

The potent capacity of the diatom *Chaetoceros muellerii* to alleviate pollution from low-density polyethylene microplastic particles (LDPE-MPs) in marine ecosystems

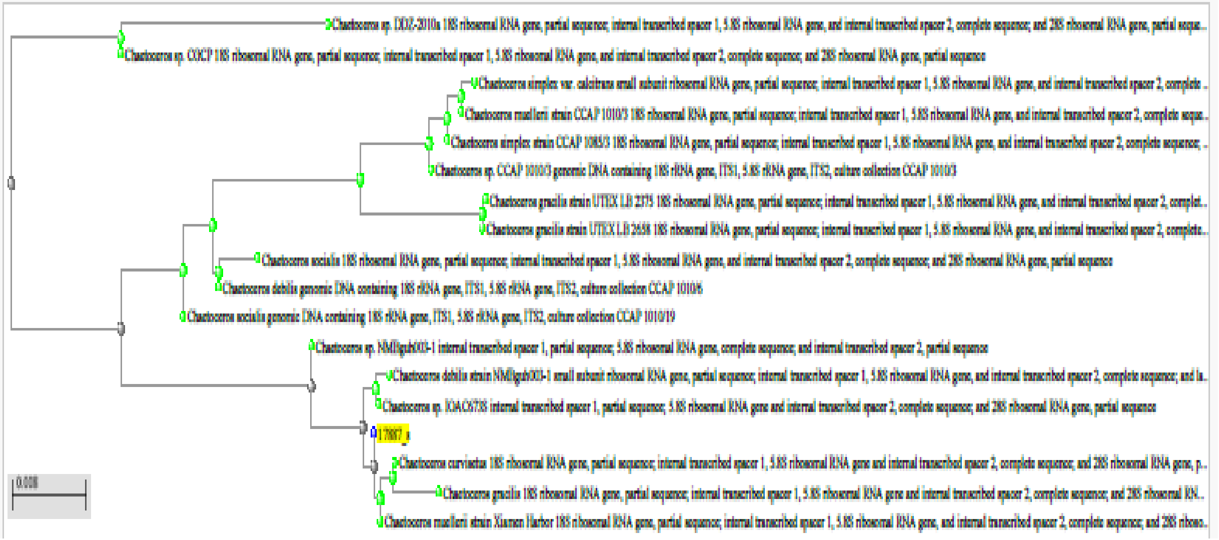

Figure 1S. A consensus phylogenetic tree based on 18S rRNA gene sequence of *Chaetoceros muellerii* strain Xiamen Harbor 18S ribosomal RNA gene with accession no. KF998567.1

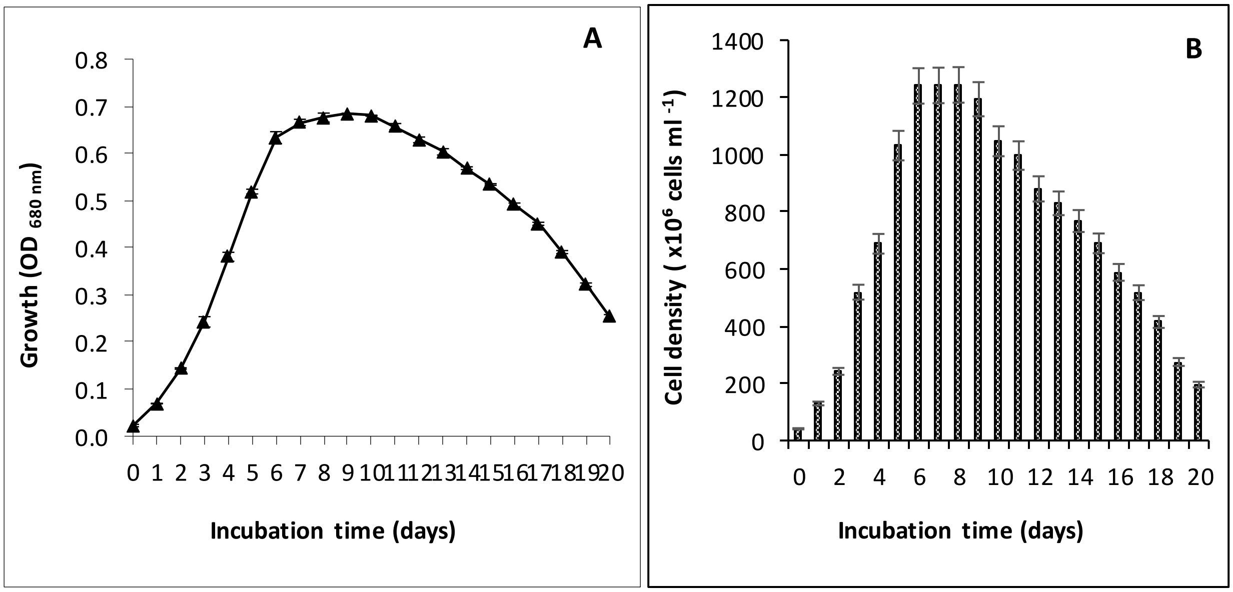

Figure 2S. Growth curve of *Chaetoceros muellerii* grown on F/2 culture medium as estimated by **A)** optical density (OD_{680}) and **B)** cell count.

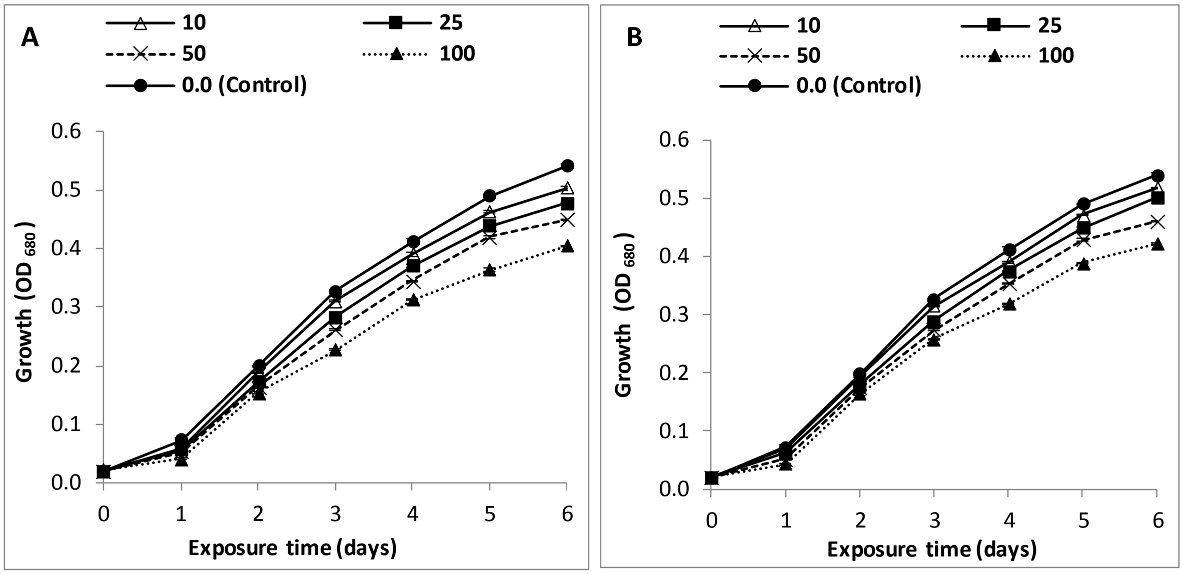

Figure 3S. Growth curves of *Chaetoceros muellerii* exposed to different concentrations (mg L^{-1}) of LDPE-MPs using particle size of 100µm (A) and 250µm (B) during 6 days of culture incubation.

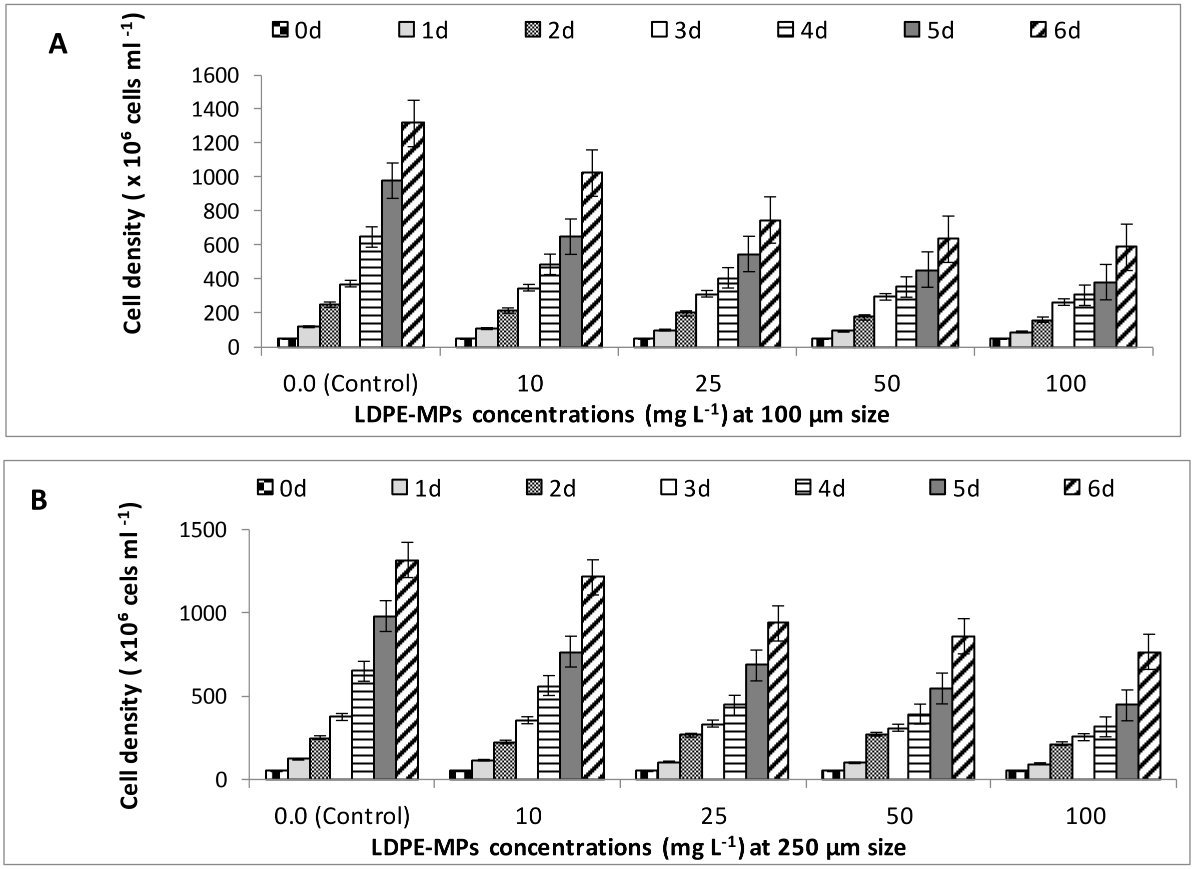

Figure 4S. Cell growth of *Chaetoceros muellerii* expressed as count (x10^6 cells ml^{-1}) after exposed to different concentrations of LDPE-MPs (mg L^{-1}) with particle size of 100µm (A) and 250µm (B) for 6 days of incubation.

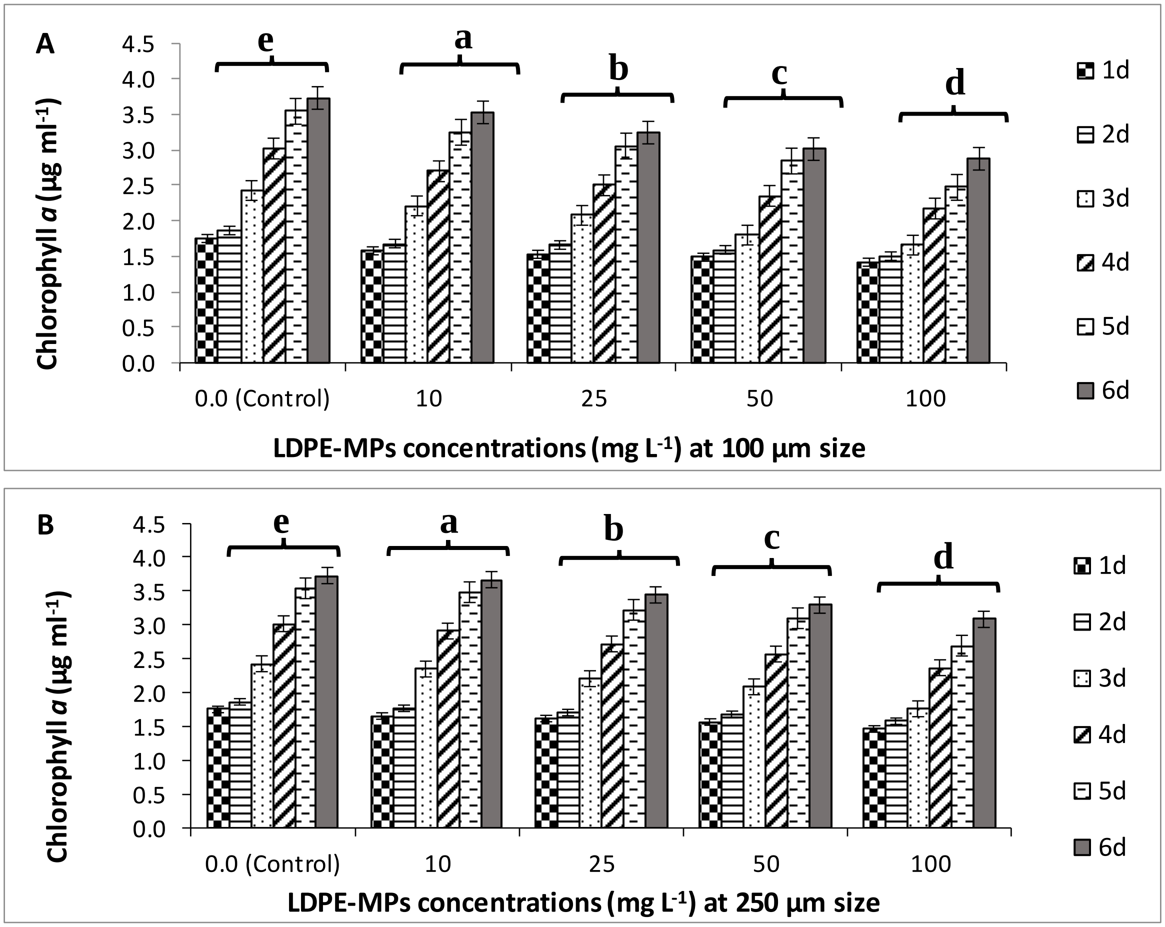

Figure 5S. Chlorophyll *a* content (µg ml^{-1}) of *Chaetoceros muellerii* cultures exposed to LDPE-MPs (mg L^{-1}) with particle sizes of 100µm (A) and 250µm (B) during 6 days of incubation. Error bars showed the SD for three replicates. Columns with different letters showed significant differences between polymer concentrations compared to the control (cultures without LDPE-MPs treatment) at $P \leq 0.05$.

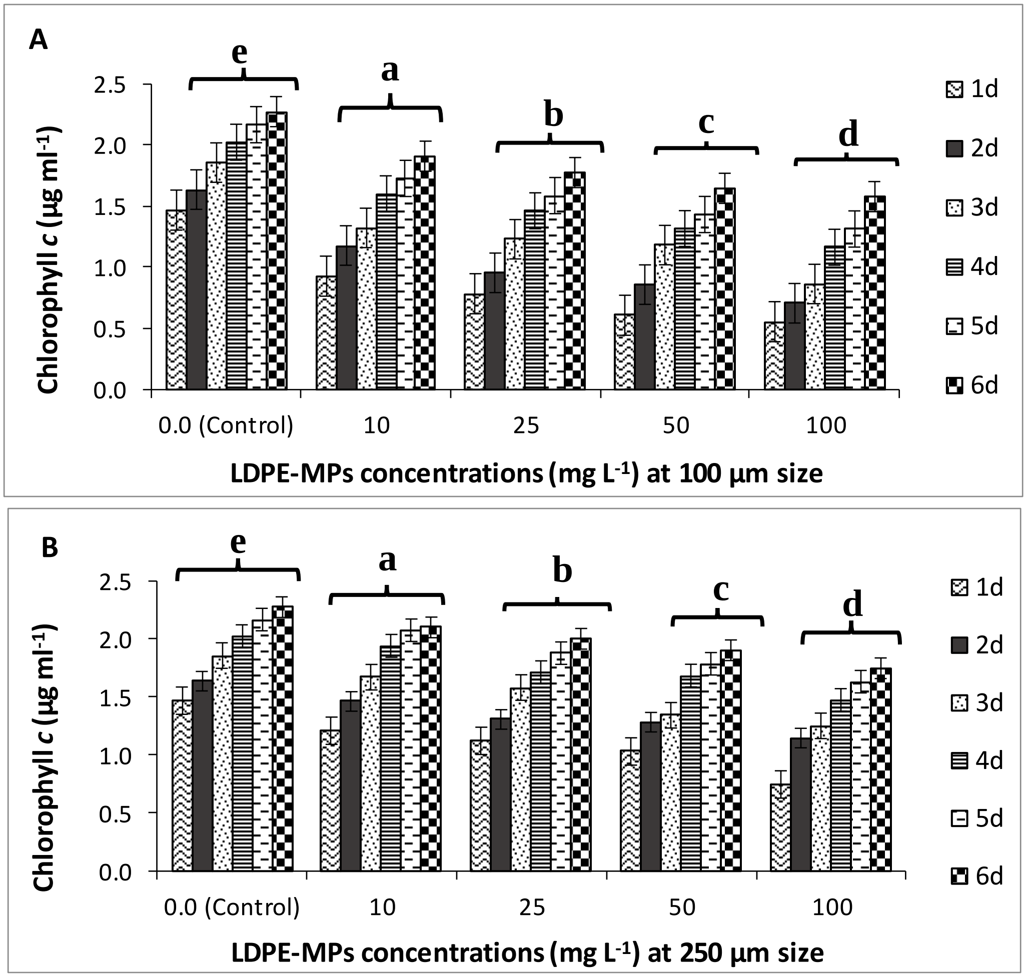

Figure 6S. Chlorophyll *c* content (µg ml^{-1}) of *Chaetoceros muellerii* cultures exposed to LDPE-MPs (mg L^{-1}) with particle sizes of 100µm (A) and 250µm (B) during 6 days of incubation. Error bars showed the SD for three replicates. Columns with different letters showed significant differences between polymer' concentrations compared to the control (cultures without LDPE-MPs treatment) at $P \leq 0.05$.

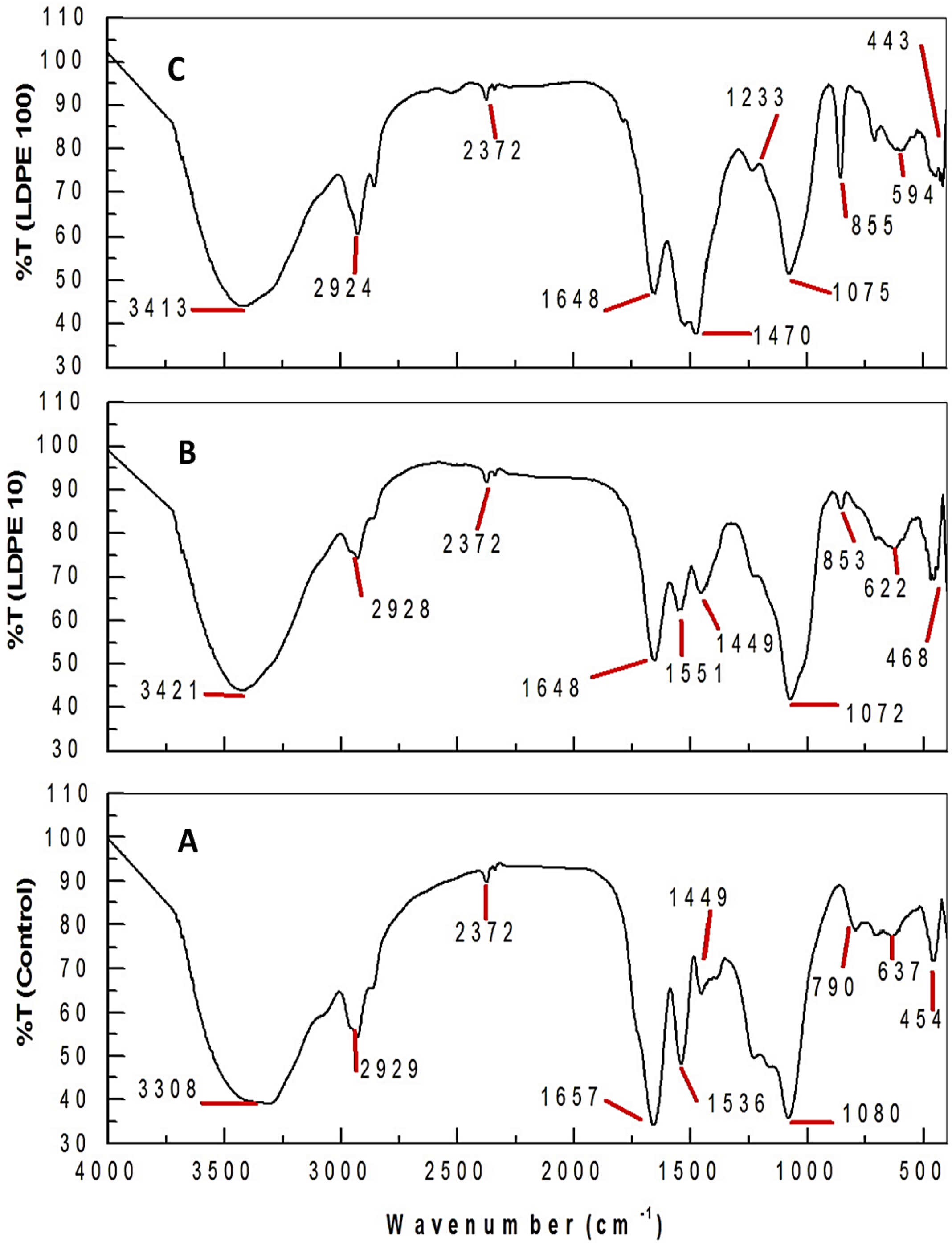

Figure 7S. FTIR comparative spectra of *Chaetoceros muellerii* cells treated with LDPE-MPs of 100 µm particle size at control (A), 10 mg L^{-1} (B), and 100 mg L^{-1} (C) concentrations.

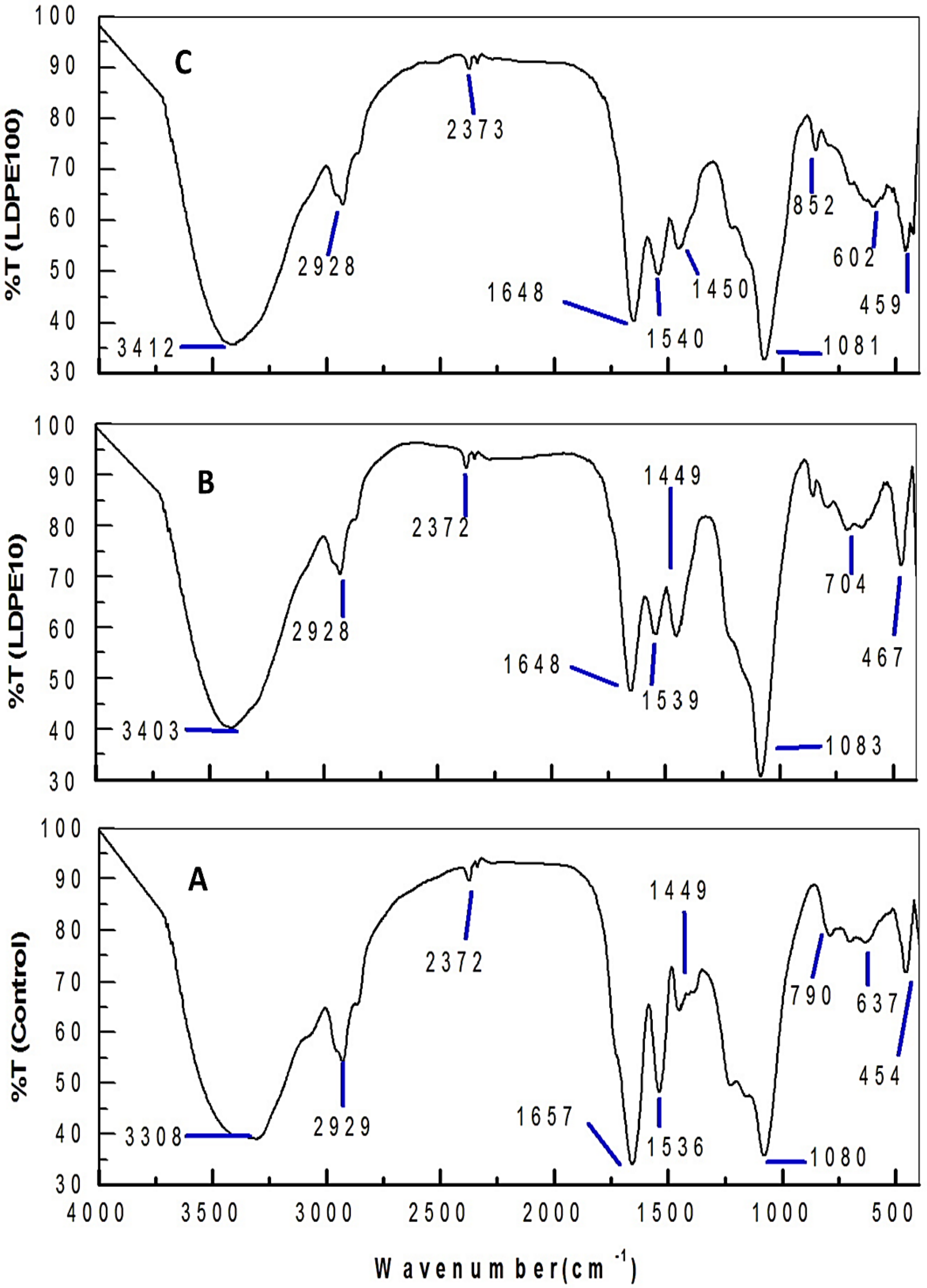

Figure 8S. FTIR comparative spectra of *Chaetoceros muellerii* cells treated with LDPE-MPs of 250 µm particle size at control (A), 10 mg L^{-1} (B), and 100 mg L^{-1} (C) concentrations.

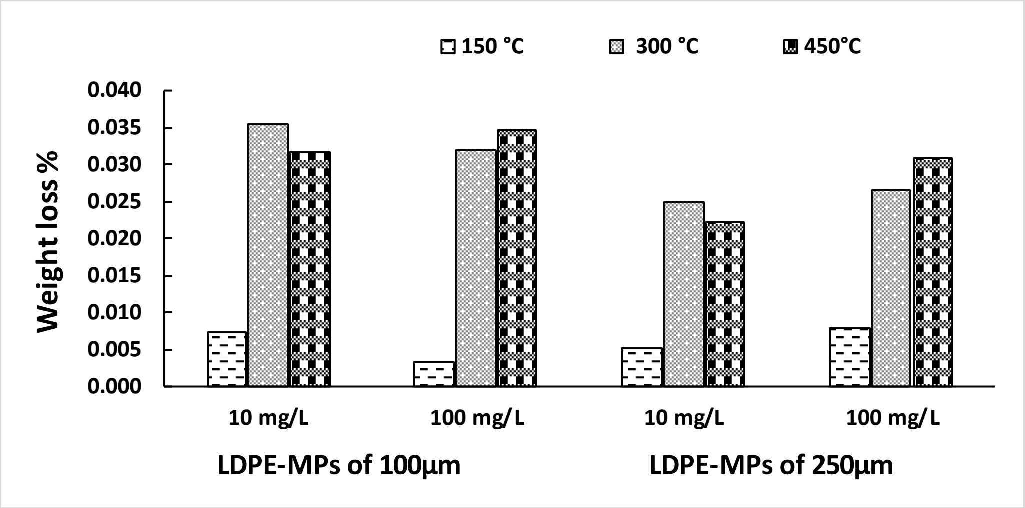

Figure 9S. Thermal analysis by combustion technique showing weight loss (%) of *Chaetoceros muellerii* cells treated with 100 and 250 µm particle sizes of LDPE-MPs as a function of the decomposition specific temperature.
